# First-Principles Microkinetic Modeling Unravelling the Performance of Edge-Decorated Nanocarbons for Hydrogen Production from Methane

**DOI:** 10.1021/acsami.2c20937

**Published:** 2023-01-26

**Authors:** Neubi F. Xavier, Glauco F. Bauerfeldt, Marco Sacchi

**Affiliations:** † School of Chemistry and Chemical Engineering, University of Surrey, GuildfordGU2 7XH, U.K.; ‡ Instituto de Química, Universidade Federal Rural do Rio de Janeiro, CEP 23890-000Seropédica, Rio de Janeiro, Brazil

**Keywords:** methane, DFT, microkinetic modeling, carbon catalysis, graphene, hydrogen production, doping, decorated edge

## Abstract

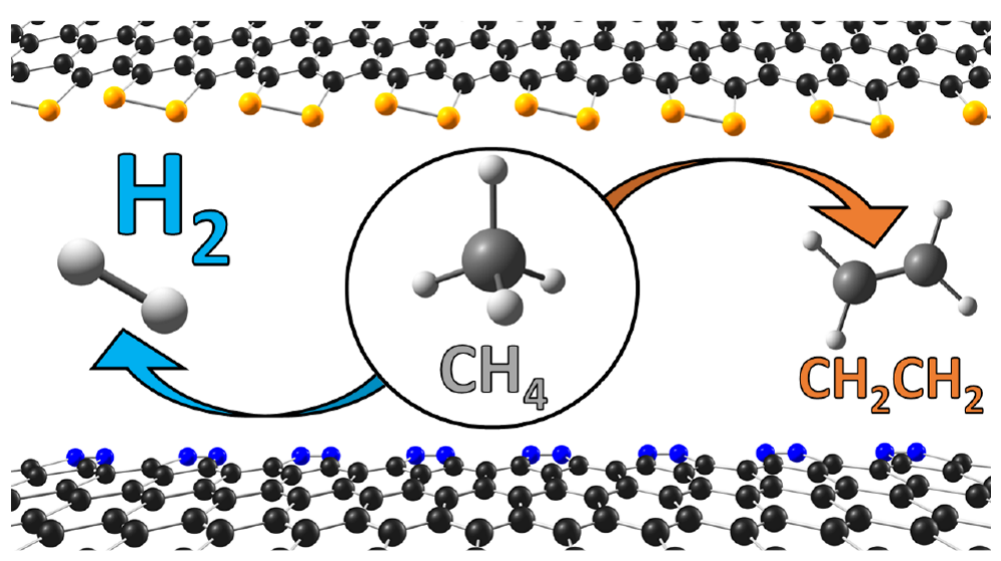

The doping of graphitic and nanocarbon structures with nonmetal atoms allows for the tuning of surface electronic properties and the generation of new active sites, which can then be exploited for several catalytic applications. In this work, we investigate the direct conversion of methane into H_2_ and C_2_H_*x*_ over Klein-type zigzag graphene edges doped with nitrogen, boron, phosphorus and silicon. We combine Density Functional Theory (DFT) and microkinetic modeling to systematically investigate the reaction network and determine the most efficient edge decoration. Among the four edge-decorated nanocarbons (EDNCs) investigated, N-EDNC presented an outstanding performance for H_2_ production at temperatures over 900 K, followed by P-EDNC, Si-EDNC and B-EDNC. The DFT and microkinetic analysis of the enhanced desorption rate of atomic hydrogen reveal the presence of an Eley–Rideal mechanism, in which P-EDNC showed higher activity for H_2_ production in this scenario. Coke deposition resistance in the temperature range between 900 and 1500 K was evaluated, and we compared the selectivity toward H_2_ and C_2_H_4_ production. The N-EDNC and P-EDNC active sites showed strong resistance to carbon poisoning, whereas Si-EDNC showed higher propensity to regenerate its active sites at temperatures over 1100 K. This work shows that decorated EDNCs are promising metal-free catalysts for methane conversion into H_2_ and short-length alkenes.

## 1 Introduction

The direct conversion of methane, CH_4_, to hydrogen (so-called catalytic methane decomposition, or CMD) is an attractive process for converting natural gas into high-value chemicals, mainly due to the cleanliness of the process^1,2^ and the large availability of natural and shale gas reserves.^3,4^ The main challenge for thermally cracking methane is the large energy required for the C–H bond cleavage;^5^ therefore, the usage of a catalyst is crucial for achieving lower temperatures for efficient conversion.^6^ The direct catalytic conversion of methane can proceed through oxidative routes, such as oxidative coupling of methane (OCM) and partial oxidation of methane (POM), and nonoxidative routes.^3,7,8^ For the former, undesirable C_2_ and C_3_ byproducts as well as CO_2_ can be formed via reactions between methyl radicals and oxygen, making the process less sustainable.^4,9^ On the other hand, the nonoxidative conversion of methane can yield high selectivity toward target products.^4,10^ This process generally requires high temperatures to overcome the kinetic barrier for methane cracking and hydrogen desorption, which can result in the severe deactivation of the catalyst due to coking, thus being a major drawback of metal-based catalysts.^1,9,11,12^ Therefore, significant effort has been made toward the design of efficient catalysts that are expected to improve the selectivity activation of C–H bonds, while being resistant to carbon poisoning.^13−16^

Much attention has been directed to the improvement of the process of catalytic methane decomposition (CMD) into hydrogen H_2_ production in the past decade.^1,9,12,14,17−20^ The benefits of the process are self-evident since the main products of CMD are pure hydrogen gas and solid carbon: CH_4_ → 2H_2_ + C.^9,18^ Turning the CMD process into a large-scale application is a top-priority because it would allow converting CH_4_ (one of the worst greenhouse gases) into hydrogen, which can be employed as a clean fuel. H_2_ is becoming ever more relevant as a promising candidate to replace fossil fuels, which are rapidly depleting and cause global warming.^2^ In fact, the major source of H_2_ production is via methane steam reforming (SMR), resulting in large emissions of greenhouse gases.^21^ Therefore, the reduction of the CO_*x*_ as a byproduct for hydrogen production is pointed to as critical to reducing costs and energy consumption.^22^

Metal-based catalysts are the most commonly employed for the CMD process, with nickel- and iron-based catalysts being the most economically viable and possessing high selectivity for hydrogen production.^1,12,15,23^ Carbon-based materials are expected to overcome the rapid deactivation due to coke deposition, which is common among metallic catalysts.^1,12,15,23^ The decomposition of methane at the gas–solid interface can be largely improved by the presence of different surface configurations on the carbon catalyst, such as defects, vacancies and low-coordination sites.^18^ In fact, the amount of defects present in the carbon surface is directly correlated to the catalytic activity for CMD,^24^ especially in the initial stages of the process.^18^ Furthermore, the catalytic activity and selectivity of graphitic structures can also be tuned by doping of metallic and nonmetallic elements, introduced during the manufacturing process. The conversion of methane with different types of carbon materials is expected to be similar in the late stages of the processes due to the formation of carbon deposits (coke) on the surface, which hinder the longevity of the carbon catalyst for hydrogen production.^25^ To the best of our knowledge, most of the current understanding of the effect of surface structure and doping on the CMD process is based on extrapolation of specific reaction steps’ findings^18,26,27^ to the overall process and studies investigating the effect of heteroatomic doping toward the nonoxidative hydrogen production from methane on carbonaceous catalysts are lacking.^15,18^ Therefore, it is mandatory that a thorough atomistic investigation of the CMD process, based on the carbonaceous catalyst structure modification, is performed in order to give insights about the main reaction mechanism, aiming at predicting intermediates’ and byproducts’ production at desired conditions, elucidating the main hydrogen formation channels and to guide the catalyst engineering.

Despite the challenges linked to the formation of single and dual active sites on graphitic structures, great advances have been achieved by adopting graphene and other nanocarbons as substrates.^28^ For example, the design and synthesis of dual active metal sites on N-doped porous carbon materials have been reported,^24^ and they showed that the doped material has vastly improved performance for methane activation.^11^ Aberration-corrected scanning transmission electron microscopy (ACSTEM) experiments reported improvements in atom-by-atom manipulation of graphitic structures, being highly relevant for the synthesis of doped materials.^29^ In this regard, the decoration of silicon on graphene edges was achieved by moving individual atoms via an electron beam, generating several configurations on graphene’s armchair and zigzag edges.^30^ Sharma et al.^31^ synthesized carbon nanotubes containing nitrogen-doped sites in the graphitic network, which showed higher activity for CO_2_ reduction at temperatures between 750 and 950 °C. Chen et al.^30^ reported the efficient formation of extended silicon Klein-type^32^ edges on graphene. Further DFT calculations predicted the high activity of the Si-decorated edges for CO_2_ reduction.^30,33,34^ Xiao et al.^35^ reported a procedure for preferential phosphorus doping on the edges of graphene layers, in which the P-doped edges showed superior catalytic activity for O_2_ evolution in comparison with pristine graphene. Dual phosphorus–nitrogen decoration showed enhanced selectivity for the nitrogen doping on graphene edges, which in turn results in higher electrochemical activity for the oxygen reduction reaction (ORR).^36^ The presence of P on the dual doping system resulted in better performance of the catalyst due to the increased charge delocalization and for promoting the N-doping at the edges of graphene, creating more active sites.^36^ Therefore, the high activity of doped edges carbon-based materials for catalytic applications has motivated studies reporting processes of guided doping^37^ and heteroatomic atom enrichment^38^ of graphene edges.

Based on the recent advances in the design and synthesis of doped graphitic nanostructures and their prospect as efficient catalysts for methane activation, in this work, we investigated the performance of edge-decorated nanocarbons (EDNCs) for the catalytic decomposition of methane into H_2_ production. We used density functional theory (DFT) calculations coupled to microkinetic modeling to elucidate the mechanism of hydrogen production over nitrogen, boron, phosphorus and silicon active sites on decorated graphene edges. The effect of pressure and surface temperature on the catalytic activity shows that the decomposition of CH_4_ over EDNCs follows a Langmuir–Hinshelwood (LH) or Eley–Rideal (ER) surface reaction mechanism.^39^ We calculated the turnover frequencies and proposed rate-determining reaction steps by means of degree of rate control (DRC) analysis. The coke resistance ability of EDNCs was evaluated by studying their selectivity for the formation of C_2_H_*x*_ and H_2_ products. Based on our calculations, the excellent catalytic performance of EDNCs is due to the balance between their capability for methane activation and active site regeneration. The very low barriers for hydrogen formation observed here provide strong evidence of the catalytic potential of EDNCs for conversion of methane into hydrogen and unsaturated hydrocarbons.

## 2 Theoretical Methods

### 2.1 Models of Edge-Decorated Nanocarbons

**Figure 1:**
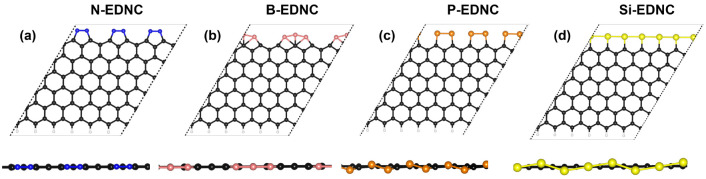
Representation of the top view (upper image) and side view (lower image) of the optimized structures of (a) N, (b) B, (c) P and (d) Si decorated zigzag edges.

The models of edge-decorated nanocarbons (EDNCs) adopted here were constructed based on recently synthesized nanocarbons with extended silicon Klein-type^32^ on armchair and zigzag graphene edges, measured at 800 °C.^30,40^ The stability of the decorated sites and their catalytic activity for the CO_2_ reduction were supported by AIMD simulations and DFT calculations.^30,33,34^ Furthermore, the presence of nitrogen, boron and phosphorus as active sites on graphene edges showed increased activity for catalytic applications.^35−38^ Based on the aforementioned reports, we studied the catalytic methane decomposition on nanocarbons edges adopting as a substrate a single-layer graphene sheet, consisting of 12 carbon atoms along the zigzag lines between the edges of the nonperiodic lattice. A similar model of the graphene catalyst was successfully employed in previous microkinetic investigations of hydrocarbon dehydrogenation on nanocarbons’ oxide edges^41^ and in the DFT investigation of the reduction of CO_2_ into C_2_ products.^34^ Therefore, a supercell consisting of a single 6 × 1 unit cell was adopted for the zigzag edges, containing 84 atoms. The zigzag edges were decorated with six nitrogen (N-EDNC), boron (B-EDNC), phosphorus (P-EDNC) or silicon (Si-EDNC) atoms. The optimized EDNC structures are shown in Figure 1. A vacuum space of 20 Å was set to avoid spurious interactions between the periodically repeated images. The bond length of X–C, in which X = N, B, P and Si, in the minimum energy structures was 1.42 Å, 1.51–1.58 Å, 1.78 Å and 1.85 Å, respectively. For the latter, a very good agreement was observed between the DFT values obtained here and the measurements of the elongated Si–C bond length on graphene of 1.82 Å, reported by Chen et al.^30^ In order to further investigate the stability of the active sites for the high-temperature methane conversion presented in this work, we performed ab initio molecular dynamics simulations for the N-EDNC, B-EDNC, P-EDNC and Si-EDNC and the details are reported in the Supporting Information. After a 5 ps simulation, the catalysts’ structures remained stable at 1000 K, proving their stability for high-temperature applications.

### 2.2 Computational Details

Plane wave, periodic spin-polarized density functional theory (DFT) calculations were carried out in the CASTEP program.^42,43^ The energy cutoff was tested and converged to a value of 550 eV, being adopted for the treatment of the valence electrons, whereas core electrons were treated by ultrasoft pseudopotentials of Vanderbilt.^44^ The electron exchange and correlation functional parametrized by Perdew–Burke–Ernzerhof (PBE)^45^ was used with the TS dispersion correction.^946^ Furthermore, the Γ-centered Monkhorst–Pack *k*-point grid was tested and converged into a mesh of 2 × 1 × 1 for the sampling of the first Brillouin zone.^46^ Transition states were located by the Linear-Quadratic-Synchronous Transit (LST/QST) algorithm^47^ and were confirmed by vibrational analysis, identifying the single imaginary frequency respective to the reaction coordinate. Overall, forces were converged to 0.025 eV Å^–1^ and tolerance for the self-consistency field calculations was set to 1 × 10^–6^ eV. Adsorption energies (*E*_*ads*_) were estimated as *E*_*ads*_ = *E*_*adsorbate*+*X*-*EDNC*_ – *E*_*X*-*EDNC*_ – *E*_*adsorbate*_, where *E*_*adsorbate*+*X*-*EDNC*_ is the total energy of the adsorbed system and *E*_*X*–*-EDNC*_ refers to energy of the bare decorated graphene edge, where X = N, B, P and Si. *E*_*adsorbate*_ stands for the energy of the adsorbate in the gas phase.

The reaction rate coefficients (*k*) for the activated chemisorption and surface reactions were calculated according to the Transition State Theory (TST).
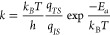
1where *h* and *k*_*B*_*T* are the Planck and Boltzmann constants, *E*_*a*_ is the activation energy and *q*_*TS*_ and *q*_*IS*_ are the partition functions for the transition state and initial state, respectively. The partition functions, as well as entropies of the adsorbates were estimated according to the lattice gas approach.^48,49^ Vibrational properties were obtained by phonons’ calculations at the Γ-point adopting the partial Hessian vibrational analysis.^50^ In this approach, the normal modes of the adsorbate and the two atomic rows closest to adsorbate (the heteroatom chain and the first carbon chain) were considered, while the remaining atoms were kept constrained. The vibrational modes with values lower than 200 cm^–1^ were assumed as 200 cm^–1^, to avoid inconsistency in the treatment of lower frequencies, in a similar approach adopted in previous works.^41,51^ The Gibbs free energy was calculated with the fundamental equation *G* = *H* – *TS*, in which *S* is the entropy and the enthalpy, *H*, comprises the DFT energy at 0 K, the zero-point energy correction and thermal contributions at a determined temperature. For the gas-phase molecule, the translational, rotational and vibrational partition functions were considered, accordingly with conventional statistical thermodynamic expressions. The Gibbs free energy of activation, Δ*G*_*a*_, was estimated as the difference between the free energies of the transition state and the initial state.

The set of ordinary differential equations (ODEs) for the coverage of species *i* was obtained as written in eq 2. The *c*_*i*,±*j*_ is the concentration of the species *i* in the elementary reaction +*j* (forward reaction) and −*j* (backward reaction). The *r*_±*j*_ refers to the rate of the respective elementary reaction. The ODEs were solved at the steady state, in which , by using the stiff solver as implemented in the MKMCXX code.^52^
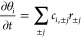
2

## 3 Results

### 3.1 Activation of CH_4_ on EDNCs

**Figure 2:**
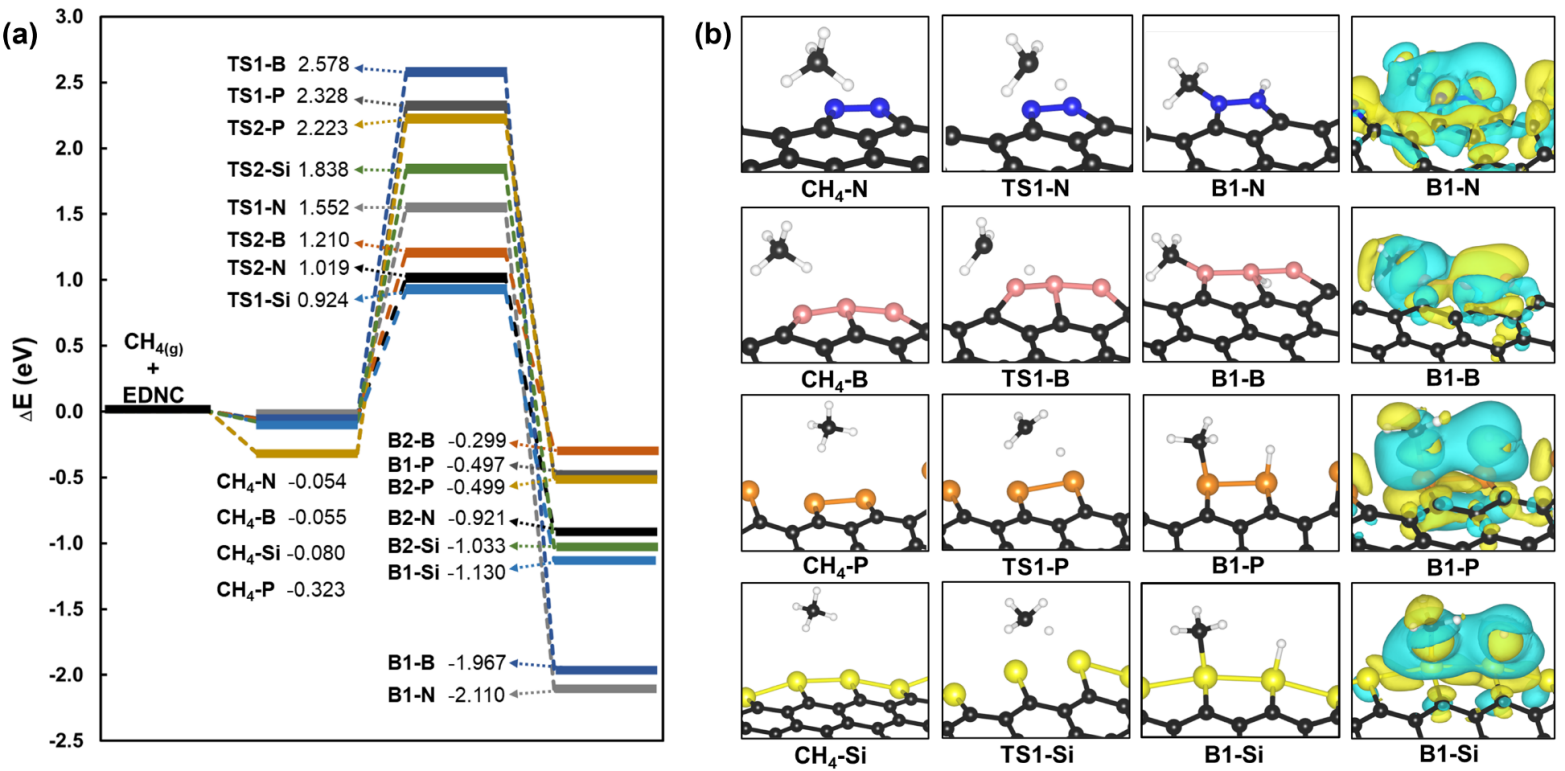
(a) Reaction energy profile for the dissociative chemisorption of methane on N-EDNC, B-EDNC, P-EDNC and Si-EDNC. The reference energy adopted as the zero value is the sum of CH_4(g)_ and the respective EDNC. (b) Representation of the stationary points for the activated methane adsorption into CH_3_ and H adsorbed on different edge atoms and their respective label adopted in this work. The charge density differences (isosurface cutoff value was 0.008 e Å^–3^) of the chemisorbed structures B1-N, B1-B, B1-P and B1–Si are depicted in the rightmost panels.

The activation of CH_4_ on the catalyst surface is the first elementary step of the whole CMD process, which can occur through a dissociative or nondissociative mechanism.^12,53^ In the first case, the cleavage of the C–H bond and adsorption occur in the same step, while in the latter the adsorption and bond cleavage occur in two different steps. The direct adsorption of methane was suggested to be more favorable in comparison to the nondissociative pathway.^11,54^ Therefore, the dissociative adsorption of CH_4_ was investigated here on the N-EDNC, B-EDNC, P-EDNC and Si-EDNC, as shown in Figure 2. First, the weak adsorption of methane on EDNCs is evidenced by the small adsorption energy obtained on N-EDNC, B-EDNC and Si-EDNC, with *E*_*ads*_ values of −0.054 eV, −0.055 eV and −0.080 eV, respectively. The strongest adsorption of CH_4_ occurs on P-EDNC, with a more negative value of −0.323 eV. The equilibrium distance adsorbed on top of the decorated heteroatom was of 3.51 Å, 3.77 Å, 3.83 Å and 3.74 Å for N-EDNC, B-EDNC, P-EDNC and Si-EDNC, which is characteristic of physisorption.

The reaction profile in Figure 2a shows the two possible outcomes of the dissociative CH_4_ adsorption: the CH_3_ radical and H end chemisorbed either on (1) different edge sites or on (2) the same edge site. For better clarity, the transition states and products are labeled by numbers 1 and 2, followed by the respective heteroatom decorating the catalyst edge (active sites N, B, P and Si). For example, in Figure 2b, the possible adsorption reactions occurring on N-EDNC proceed from the physisorbed CH_4_ (CH_4_-N), going through the transition state, TS1-N, and forming CH_3_ and H anchored on two N sites (B1-N). Chemisorption pathways occurring on distinct edge atoms, for N-EDNC, B-EDNC, P-EDNC and Si-EDNC, are presented in Figure 2, whereas chemisorption processes occurring on the same decorated atom are presented in Figure S2 in the Supporting Information.

The chemisorption of CH_3_ with an in-plane configuration was obtained on N-EDNC and B-EDNC, whereas results showed the methyl positioned in an out-of-plane orientation over the decorated edges on P-EDNC and Si-EDNC (Figure 2b). The minimum energy configurations of the hydrogen atoms chemisorbed on the decorated edges are either with the H positioned in the same plane of the edges (N-EDNC), with the H adsorbed in an out-of-plane bridge configuration between two boron atoms (B-EDNC) or with H anchored on the top of the active site in an out-of-plane configuration (P-EDNC and Si-EDNC) (Figure 2b). The most kinetically favorable chemisorption pathway occurred on the Si-EDNC catalyst, with a barrier height of 0.92 eV (TS1-Si), resulting in CH_3_ and H anchored on two Si atoms (B1–Si, Figure 2), being 0.91 eV lower than TS2-Si. On the other hand, the dissociative chemisorption of methane on the same edge atom was more kinetically feasible on N-EDNC, B-EDNC and P-EDNC, following the order: TS2-N (1.01 eV) < TS2-B (1.21 eV) < TS1-N (1.55 eV) < TS2-P (2.22 eV) < TS1-P (2.32 eV) < TS1-B (2.57 eV).

It is possible to observe from Figure 2a that the adsorption of methane is an exothermic process for all the decorated EDNCs considered in this study, leading to more thermodynamically stable products when CH_3_ and H are chemisorbed on different edge sites. It is noteworthy that the CH_4_ adsorption was reported to be endothermic on doped metal sites on graphene^11,55^ and bimetallic catalysts^54,56^ in previous works. A large energetic difference was found between the two adsorption configurations of CH_3_ and H on N-EDNC and B-EDNC. The B1-N and B1-B were 1.18 and 1.67 eV more stable than B2-N and B2-B, respectively. The adsorption of methane on two edge sites was more energetically similar among the EDNCs in comparison with the chemisorption of methyl and hydrogen on the same edge site. In this case, B2-P was only 2 meV more stable than B1-P while B1-Si was 97 meV more stable than B2-Si. The adsorption of the H atom and CH_3_ is overall more favorable on N-EDNC and B-EDNC edges, as reported in Table S1, with N-EDNC having *E*_*ads*_ of −2.21 eV and −1.14 eV, respectively, whereas *E*_*ads*_ values of −0.63 eV and −0.20 eV were obtained for the adsorption of H and CH_3_ on P-EDNC.

In general, hydrogen atoms interact more strongly with the decorated edges rather than methyl, with Δ*E*_*ads*_ being roughly 5% to 200% more negative on B-EDNC and P-EDNC, respectively, as evidenced in Table S1. To better elucidate this, the interplay of charges in the chemisorption process was investigated by mapping the Mulliken charges’ density difference for the most thermodynamically stable products, as reported in Figure 2b. A charge accumulation occurred in the C–X and H–X bonds region (X = N, B, P and Si), whereas depletion of charges was observed in the interface region. A quantitative analysis of the atomic charge population with respect to each edge atom is presented in Table S2. A decrease of −0.182 e and −0.190 e of the Mulliken charge was observed after the chemisorption of CH_3_ on N and B sites, respectively, suggesting a higher electron density around the chemisorbed hydrogen in comparison with P-EDNC and Si-EDNC

### 3.2 Reaction Mechanisms of H_2_ Formation on EDNCs

**Figure 3:**
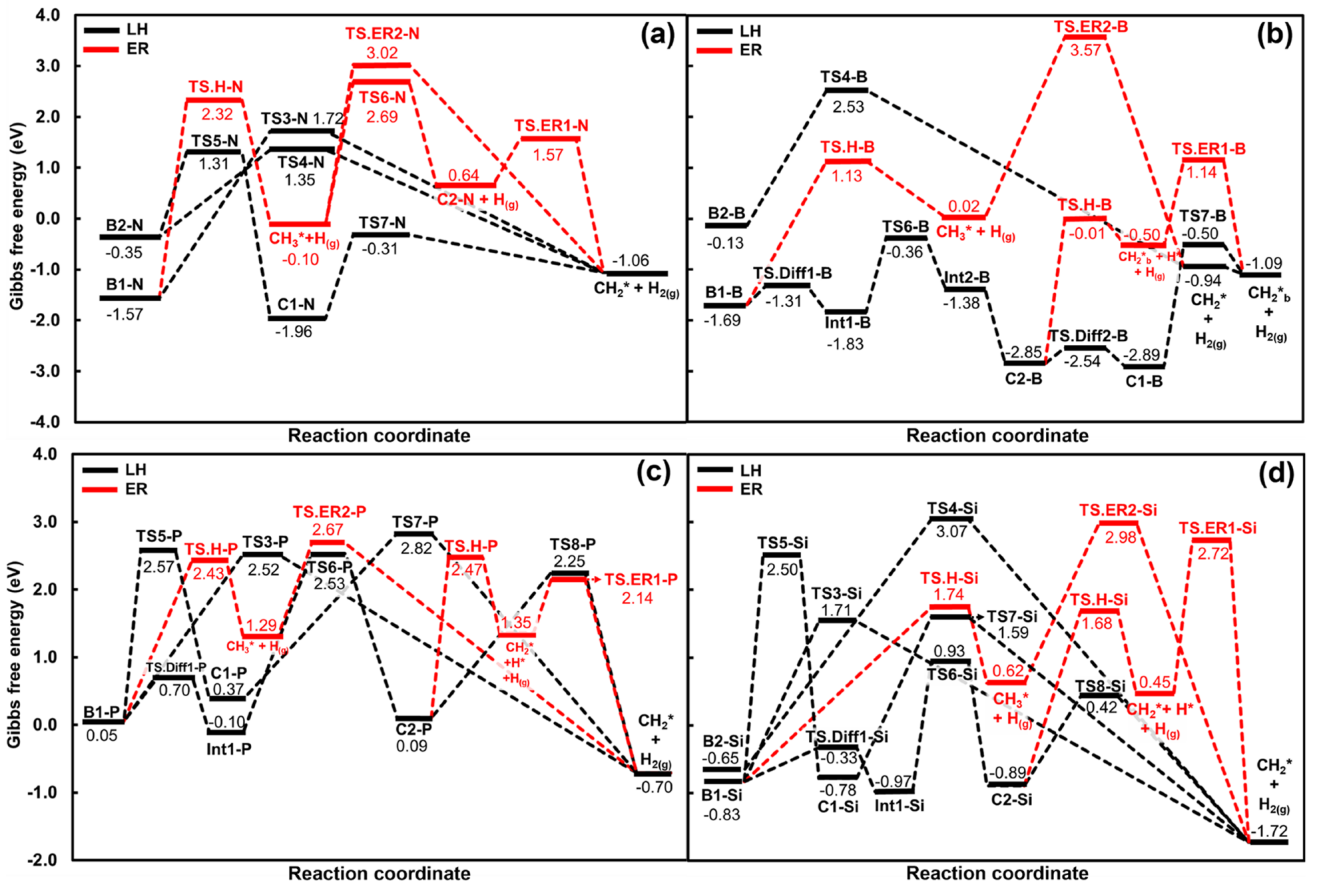
Gibbs free energy reaction profiles of the H_2_ formation on (a) N-EDNC, (b) B-EDNC, (c) P-EDNC and (d) Si-EDNC at 1000 K, 1 bar. The reference for the zero-energy value was adopted as the physisorbed CH_4_. LH and ER stand for Langmuir–Hinshelwood and Eley–Rideal reaction mechanisms, respectively.

Next, we study the pathways of H_2_ formation on EDNCs, by systematically investigating eight possible reaction channels from the chemisorbed methyl and atomic hydrogen on edges at the reaction condition of 1000 K. This temperature was adopted due to evidence of high conversion of methane at temperatures higher than 1000 K.^11,12,57−59^ The labels TS3 and TS4 describe the transition states of H_2_ formation channels from B1 and B2 products, respectively. Hereafter, the star index “*” indicates when the species is adsorbed on the EDNC, whereas its absence represents gas-phase compounds. In these pathways, the formation of hydrogen molecules proceeds through the concomitant cleavage of the C–H bond from the methyl and the desorption of H from the respective edge site. Besides the direct H_2_ formation from B1, the deprotonation of CH_3_* is possible to occur either by forming CH_2_* and a hydrogen adatom (hereafter referred as HH*, i.e. two hydrogen atoms chemisorbed to the same active site) or by the chemisorption of hydrogen in a different site, resulting in CH_2_* and 2H*. The transition states for the two deprotonation reactions were labeled as TS5 and TS6, respectively, and their products were defined as C1 and C2. The same consideration was applied for the H_2_ formation from HH* and from 2H*, being named TS7 and TS8, respectively. The transition states relative to the diffusion of H* on edges (labeled as TS.Diff) were included in the mechanism and discussed throughout the manuscript. The free energy diagrams for the mechanism of H_2_ formation on N-EDNC, B-EDNC, P-EDNC and Si-EDNC, at 1000 K, are presented in Figure 3 and the structures and labels of the minimum energy intermediates and transition states for all the elementary steps are shown in Figures S2–S5, respectively.

The desorption of H and H_2_ from carbonaceous surfaces can occur through classical and quantum reaction routes.^60^ The recombination of H into H_2_ through a Langmuir–Hinshelwood mechanism might become more favorable on graphene edges,^61^ whereas Han et al.^60^ suggested that the rate of H_2_ formation over graphitic structures is likely affected by the presence of quantum tunnelling effects, which increase the desorption of H from the graphene/graphite surface. In this respect, we have investigated the presence of gas-phase atomic hydrogen with variable partial pressure. We located transition states for the reaction channels of H_2_ formation by the reaction of gas-phase H atoms impinging on the chemisorbed H* or CH_3_* fragments, in an Eley–Rideal (ER) mechanism, labeled as TS.ER1 and TS.ER2, respectively. The saddle points for hydrogen desorption are labeled TS.H in Figure 3.

The deprotonation of CH_3_* on the N-EDNC, forming an adatom (C1-N), is more kinetically favorable than the direct formation of H_2_ from B1-N by 0.41 eV. However, the formation of molecular hydrogen from HH* occurs through TS7-N with a Δ*G*_*a*_ of 1.65 eV. The reaction pathways occurring on N-EDNC are limited by the occupation of the two N active sites on B1-N, after the dissociative adsorption of CH_4_ (Figure S3); thus, the deprotonation reaction CH_3_* → TS6-N → C2-N can only proceed after the desorption of atomic hydrogen (H* → TS.H-N → H_(g)_, Δ*G*_*a*_ = 3.89 eV), in which an additional N active site becomes available. The deprotonation of CH_3_*, through TS6-N, to the N site is 0.33 eV more favorable than the competitive reaction H_(g)_ + CH_3_* → TS.ER2-N → CH_2_* + H_2(g)_, indicating that the formation of CH_2_* and H* on the N active sites is preferable over the molecular hydrogen formation through TS.ER-2, when H_(g)_ is available. On the other hand, after the formation of C2-N, a Δ*G*_*a*_ of only 0.93 eV is expected for the molecular hydrogen formation reaction H* + H_(g)_ →TS.ER1-N → H_2(g)_. The most kinetically favorable reaction path of methane adsorption leads to B2-N, which has a direct pathway of H_2_ formation through TS4-N, with a free energy barrier of 1.66 eV.

It is noteworthy that B-EDNC has a row of three close B active sites on edges, which is more energetically stable than a configuration with two B atom rows on edges (Figure S4). A steric hindrance between adjacent chemisorbed species was observed, making it difficult for the H* and CH_3_* to further react and form H_2_; therefore, we investigated alternative pathways for this reaction. The diffusion of H* to the B site opposite from CH_3_* (B1-B → TS.Diff_1_-B → Int1-B) is more favorable, with a Δ*G*_*a*_ of 0.38 eV (Figure 3). The deprotonation CH_3_* → TS6-N → C2-N occurs with a Δ*G*_*a*_ of 2.63 eV resulting in CH_2_* and H* chemisorbed on the same B site, with the latter in a bridge configuration (Int2-B). Investigating the formation of 2H* (C2-B) and HH* (C1-B), alongside CH_2_*, allows us to conclude that a reconstruction occurs on the B-EDNC in which CH_2_* is bonded with two rows of B sites (Figure S4), leading to a more thermodynamically stable structure (from −1.38 eV to −2.85 eV). In Figure 3b, the CH_2_ bridged with two rows of B sites is labeled as CH_2_*_b_. From C2-B, a diffusion of the H* can occur, forming the hydrogen adatom (C2-B → TS.Diff2-B → C1-B) with a low Δ*G*_*a*_ of 0.31 eV, from which H_2_ is formed, passing through TS7-B, with a Δ*G*_*a*_ of 2.39 eV. The atomic H can desorb from C2-B with an activation free energy of 2.84 eV, followed by an ER reaction to H_2_ and CH_2_*_b_ formation, through TS.ER1-B, with a Δ*G*_*a*_ of 1.64 eV. A direct pathway from B2-B through TS4-B, leading to H_2_, is expected to occur with a free energy barrier of 2.66 eV. The ER reaction CH_3_* + H_(g)_ → TS.ER2-B, leading to CH_2_* and H_2(g)_, proceeds through a higher Gibbs free energy of activation (3.57 eV) and is the less favorable channel of H_2_ formation on B-EDNC.

The edges of P-EDNC and Si-EDNC are similarly arranged in a complete zigzag chain of atoms (Figure 1). In this way, the mechanisms of H_2_ formation on these two nanocatalyst edges are expected to be similar. Overall, the Gibbs free energy of activation for the direct channels of H_2_ formation on Si-EDNC are the highest of the EDNCs, which can be attributed to the steric hindrance when species are chemisorbed on adjacent Si sites (Figure S6), leading to a slight rearranging of the silicon atom chain. This hindrance is less prominent in P-EDNC (Figure S5), in which the P atoms are arranged in pairs, such as in the N-EDNC, although being closer to allow the interactions among adjacent P atom pairs. The diffusion of hydrogen between P or Si sites is faster than the competitive reactions from B1, i.e., the direct H_2_ formation (B1 → TS3 → CH_2_* + H_2(g)_), the adatom formation (B1 → TS5 → C1) and the atomic hydrogen desorption, as shown in Figure 3. The TS.Diff1-P and TS.Diff1-Si showed activation free energies of 0.65 and 0.50 eV, respectively.

The reaction channel B1-P → TS3-P → CH_2_* + H_2(g)_ proceeds through a Δ*G*_*a*_ of 2.47 eV. The only pathway from B2-P is the interconversion to the more stable B1-P, which occurs over a small diffusion barrier (TS.Diff2-P, Δ*E* = 0.69 eV), from which it can be assumed that only B1-P is present at higher temperatures. Other reactions starting from B1-P and passing through TS5-P, TS.H-P, as well as the CH_3_ deprotonation to the P site (Int1-P → TS6-P → C2-P) and H_2_ formation from HH* (C1-P → TS7-P → H_2(g)_) and 2H* (C2-P → TS8-P → H_2(g)_) have close Δ*G*_*a*_ values of 2.52 eV, 2.38 eV, 2.63 eV, 2.47 eV and 2.16 eV, respectively. The Eley–Rideal reactions are more kinetically favorable for H_2_ formation on P-EDNC, showing energy barriers of 0.79 and 1.38 eV for the H* + H_(g)_ → TS.ER1-P → H_2(g)_ and CH_3_* + H_(g)_ → TS.ER2-P → H_2(g)_ reaction steps, respectively.

With respect to Si-EDNC, the direct pathways for H_2_ formation, B1-Si → TS3-Si → H_2(g)_ and B2-Si → TS4-Si → H_2(g)_, need to overcome Δ*G*_*a*_ values of 2.54 and 3.15 eV, respectively. The CH_3_* deprotonation steps are expected to proceed through TS5-S1 and TS6-Si with a Δ*G*_*a*_ of 3.33 and 1.90 eV, respectively. The hydrogen atoms chemisorbed on Si sites are expected to recombine into H_2_ with roughly the same Δ*G*_*a*_ values for C1-Si → TS7-Si → H_2(g)_ and C2-Si → TS.ER2-P → H_2(g)_ of 2.37 and 2.36 eV, respectively. The desorption of H from the Si site occurs after overcoming a Δ*G*_*a*_ of 2.57 eV from B1-P. Overall, the H_2_ formation from the ER mechanism CH_2_* + H_(g)_ →TS.ER1-Si → H_2(g)_ is the most favorable molecular hydrogen reaction channel on Si-EDNC with a Δ*G*_*a*_ of 2.27 eV (CH_2_* + H_(g)_ →TS.ER1-Si → H_2(g)_).

### 3.3 Catalytic Activity of EDNCs for H_2_ Formation

**Figure 4:**
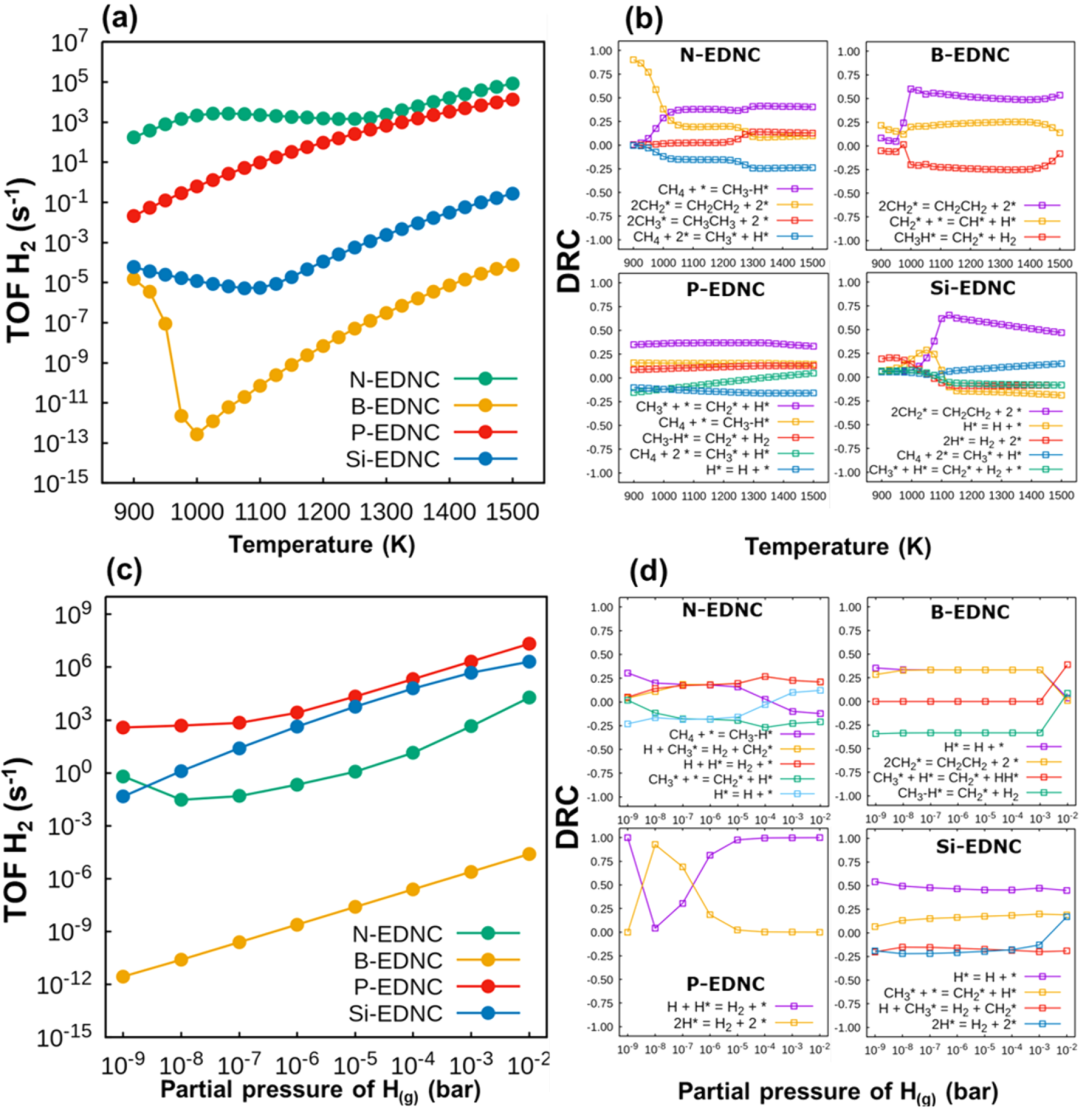
Turnover frequencies of H_2_ formation (a) against temperature and (c) partial pressure of atomic hydrogen on EDNCs. Degree of rate control on N-EDNC, B-EDNC, PEDNC and Si-EDNC as a function of (b) temperature and (d) partial pressure of H. The total pressure was set constant at 1.0 bar. The reactions with small contributions to the global rate coefficients at the entire temperature range were omitted for better clarity.

To determine the most favorable reaction pathway on different EDNCs, we performed microkinetic modeling simulations considering the individual reactions. A reaction network consisting of 14 elementary steps was built, and Arrhenius parameters for each step on N-EDNC, B-EDNC, P-EDNC and Si-EDNC are shown in Tables S3–S6, respectively. For the sake of clarity, we have adopted the nomenclature previously described for the labeling of the transition states, to identify each reaction included in the microkinetic modeling (e.g. TS1-N refers to the transition state of reaction R1-N). The formation of C2 products on nitrogen-doped graphene through the nonoxidative conversion of methane has been previously investigated and suggested as a promising catalytic route.^11^ Therefore, the coupling of methyl groups into ethene (R9: CH_2_* + CH_2_* → CH_2_CH_2_ + 2*) and ethane (R10: CH_3_* + CH_3_* → CH_3_CH_3_ + 2*) was included in the microkinetic simulations, since CH_3_* and CH_2_* are the main products of methane adsorption and H_2_ production, respectively. High coverage of either CH_3_* or CH_2_* is expected depending on the kinetic favorability among the CH_4_ chemisorption, CH_3_* deprotonation and direct pathways of H_2_ formation from CH_3_* and H*; thus, R9 and R10 are related to the regeneration of the catalytic sites. Furthermore, we have included in the simulations the step corresponding to the further decomposition of CH_2_ on EDNC, R11: CH_2_* → CH*. The turnover frequencies (TOFs)^62^ for H_2_ formation on N-EDNC, B-EDNC, P-EDNC and Si-EDNC, as a function of temperature and partial pressure of atomic hydrogen are shown in Figure 4a and Figure 4c, respectively.

**Figure 5:**
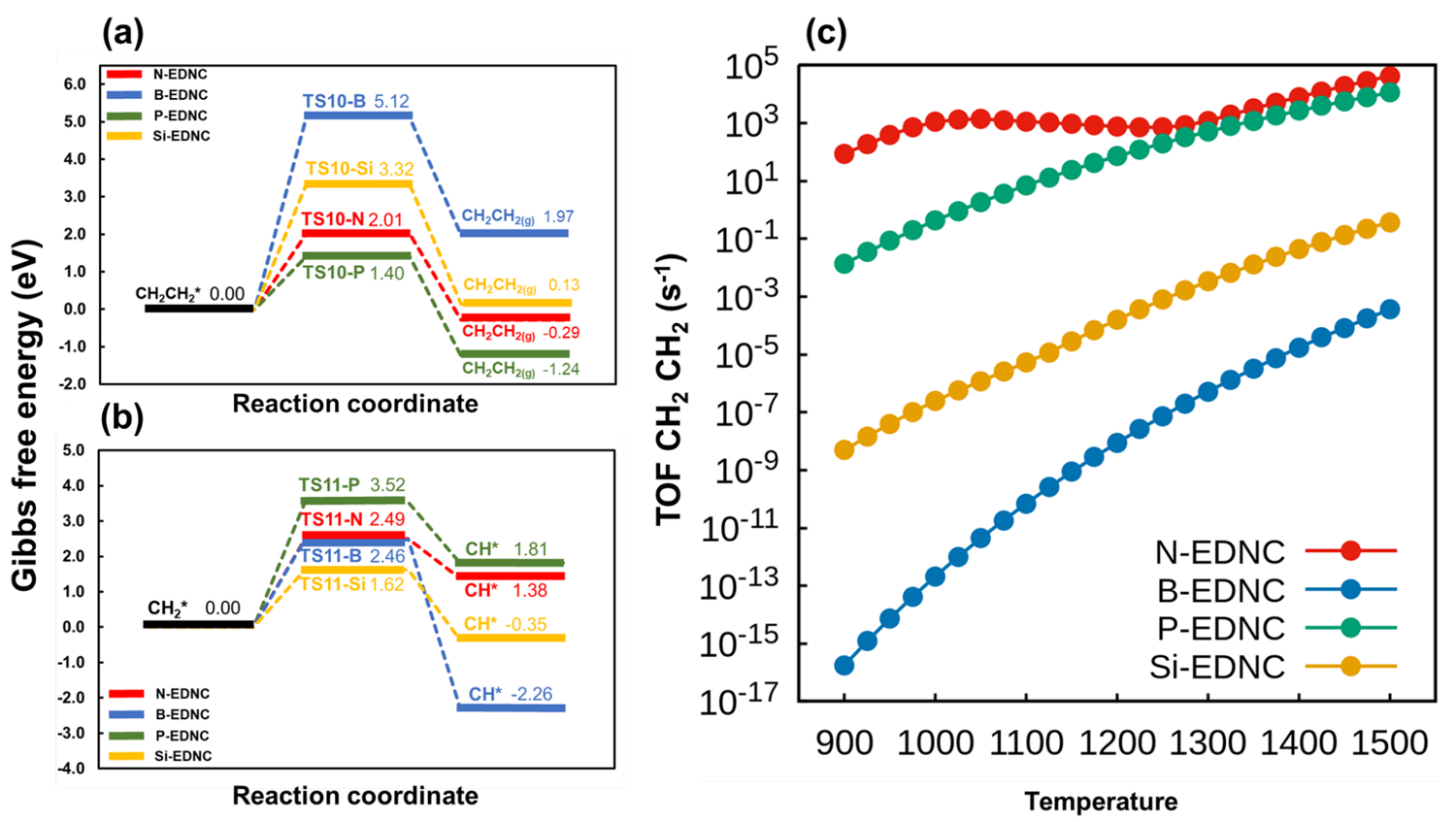
Gibbs free energy reaction profile at 1000 K, 1 bar, for the (a) formation of CH_2_CH_2_ and (b) decomposition of CH_2_* into CH*. (c) Turnover frequencies of CH_2_CH_2_ formation against temperature.

The predicted H_2_ formation TOF (Figure 4a) on N-EDNC is at least 2 orders of magnitude higher than those of the other EDNCs, in the temperature range between 900 and 1300 K, being the most efficient catalyst. At temperatures higher than 1300 K, the TOF difference between N-EDNC and P-EDNC decreases, reaching the same order of magnitude. The Si-EDNC activity on hydrogen production diminished with increasing temperature until 1100 K. The TOF on B-EDNC drastically decreases in the temperature range between 900 and 1000 K and consistently increases at the entire range between 1000 and 1500 K; however, this EDNC is expected to be much less active for H_2_ production than other EDNCs, with TOF values several orders of magnitude lower. Further investigations were made to analyze the effect that each elementary step has in the global reaction rate on each catalyst. To this end, we calculated the degree of rate control coefficient, DRC, (eq 3)^63^ for reaction step *i*, as a function of temperature
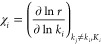
3where χ_*i*_ stands for the degree of rate control coefficient, *r* is the overall rate coefficient, and the equilibrium constant, *K*_*i*_, is the ratio of the forward (*k*_*i*_^+^) and reverse (*k*_*i*_^–^) rate constants. From the results, reported in Figure 4b, we observe that the TOF of H_2_ formation between 900 and 1000 K is controlled by the direct CH_2_CH_2_ formation on N-EDNC, which corresponds to a Δ*G*_*a*_ of 2.40 eV (free energy reaction profile for ethene formation, at 1000 K, is reported in Figure 5a, and transition state geometries are shown in Figure S7). At temperatures higher than 1000 K, the adsorption of methane into CH_3_-H* (Δ*G*_*a*_ = 1.27 eV) has the highest rate contribution. This goes in agreement with the surface species coverage as a function of temperature, reported in Figure S8, in which the coverage of CH_2_* decreases while ethene production is dominant and the adsorption of methane proceeds in the regenerated free sites. The opposite is observed in B-EDNC, in which the formation of ethene is the rate-determining step at temperatures higher than 1000 K (Figure 4b). This is correlated with the active sites on B-EDNC being difficult to be recovered due to the need to overcome a very high Δ*G*_*a*_ for CH_2_CH_2_ production of 5.12 eV (Figure 5a), confirmed by the decreasing of the CH_2_* coverage and with the increasing coverage of CH* on the catalytic surface sites as a function of the temperature increase (Figure S8). The formation of CH* from CH_2_ is more facile in comparison with the CH_2_* coupling into CH_2_CH_2_, due to having a lower Δ*G*_*a*_ of 2.46 eV (Figure 5b). In respect to P-EDNC, the migration of hydrogen from CH_3_* to the edges has the highest DRC value at the entire temperature range, in agreement with the decreasing of the coverage of CH_3_* and with the increase of availability of reactive sites on P-EDNC (Figure S8), due to the reaction of ethene production being faster (Δ*G*_*a*_ = 1.40 eV, Figure 5a) than the adsorption of methane into B1-P on P-EDNC (Δ*G*_*a*,*R1-P*_ = 2.57 eV). On Si-EDNC, the formation of H_2_ due to association of 2H* and the hydrogen atom controls the reaction rate between 900 and 1100 K, being reduced at this temperature range. The decrease in the contribution of the reaction H* + H* → H_2_ in the global rate is reflected in the drop of the TOF of H_2_, on Si-EDNC, at the same temperature range (Figure 4a). At temperatures higher than 1100 K, the coupling of CH_2_ into ethene (R9-Si: 2CH_2_* → CH_2_CH_2(g)_) has the highest DRC coefficient, which is related to the much higher Δ*G*_*a*_ of 3.36 eV, in comparison with the conversion of CH_2_* into CH* (Δ*G*_*a*,*R11*-*Si*_ = 1.62 eV). Consequently, there is complete coverage of CH* on the reactive sites of Si-EDNC (Figure S8). The free energy reaction profiles at 1000 K for the reaction CH_2_* → CH* on N-EDNC, B-EDNC, P-EDNC and Si-EDNC are reported in Figure 5b, and the geometries of stationary points are reported in Figure S9. It is noteworthy that the formation of CH_3_CH_3_ on EDNCs proceeds over a high Δ*G*_*a*_ ranging from 4.31 to 5.31 eV (Figure S10); therefore, a negligible concentration of ethane was observed for all substrates and conditions.

We investigated the effect of the increasing amount of atomic hydrogen on the overall rate coefficient by gradually increasing its partial pressure, *P*_*H*_, during microkinetic simulations, at a fixed temperature of 1000 K (Figure 4c). The H_2_ formation TOF on P-EDNC is the highest in the entire range of H partial pressure investigated (10^–9^ bar to 10^–1^ bar). At higher partial pressure of atomic hydrogen, the TOF on Si-EDNC increases, reaching values 1 order of magnitude lower than those of P-EDNC. On the other hand, the H_2_ TOF on N-EDNC decreases at *P*_*H*_ values between 10^–9^ and 10^–7^ bar, and it is lower than the H_2_ TOF on Si-EDNC and P-EDNC in the entire *P*_*H*_ range studied. For *P*_*H*_ values higher than 10^–6^ bar, the H_2_ TOF on P-EDNC and Si-EDNC converges to roughly the same order of magnitude. The lowest activity for H_2_ formation was observed on B-EDNC. The superior performance of P-EDNC and Si-EDNC is elucidated on the DRC coefficient investigation as a function of *P*_*H*_, reported in Figure 4d. The formation of H_2_ dominates the global rate reaction on P-EDNC, alternating between the Eley–Rideal reaction, H* + H_(g)_ →H_2(g)_ (Δ*G*_*a*,*R*.*ER1*-*P*_ = 0.79 eV) and 2H* → H_2_ + 2* (Δ*G*_*a*,*R8*-*P*_ = 2.16 eV), with the former being the rate-determining step in higher *P*_*H*_ values. This is explained by kinetic favorability of the of H_(g)_ adsorption, which is competitive to R.ER1-P, with a low Δ*G*_*a*_ of 1.14 eV. A similar conclusion can be made in the case of N-EDNC, in which the ER mechanisms have the highest DRC coefficients at the increasing *P*_*H*_. On the Si-EDNC the desorption of atomic hydrogen controls the overall rate coefficient due to its higher free energy barrier of 2.75 eV in comparison with competitive reactions. The performance of B-EDNC for H_2_ formation is the worst among other nanocarbons studied here, due to the high barriers for the production of ethene (Δ*G*_*a*,*R9*-*B*_ = 5.12 eV) and for hydrogen desorption (Δ*G*_*a*,*R*.*H-B*_ = 2.57 eV), with both reactions having the major contribution to the overall rate at the entire pressure range.

### 3.4 Selectivity to H_2_ and C_2_H_4_ Products and Coke Deposition Resistance on EDNCs

As stated in the previous section, the reactions of ethene formation (R9: CH_2_* + CH_2_* → CH_2_CH_2_ + 2*) and ethane formation (R10: CH_3_* + CH_3_* → CH_3_CH_3_ + 2*) were investigated as possible catalyst regeneration steps, with the former being the most kinetically favorable active site regeneration reaction. The viability of the regeneration step can be observed by analyzing the turnover frequencies of CH_2_CH_2_ formation, as shown in Figure 5c, from which the TOF of ethene is roughly the same order of magnitude than the H_2_ TOF at the entire temperature range on N-EDNC and P-EDNC. However, the high temperatures of over 1000 K in which the CMD process is conducted make the catalyst susceptible to coke deposition, leading to the deactivation of the reactive sites.^1,11,12,55,59,64^ Therefore, we investigated the resistance to coking of the active sites on the EDNCs by evaluating the dehydrogenation from CH_2_* to CH*, as it was shown to be the rate-determining step of the methane decomposition in previous works^55,65,66^ and is being applied for investigations of carbon formation on the catalysts. Furthermore, the selectivity between CH* and CH_2_* is pointed as crucial for determining the efficiency of the catalytic formation of C2 products.^67^ We calculate the selectivity of products’ formation (*s*_*i*_, eq 4), i.e, the ratio of the rate of product formation (*r*_*i*_) over the rate of the three main products: CH*, CH_2_CH_2_ and H_2_, as a function of temperature.
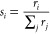
4

**Figure 6:**
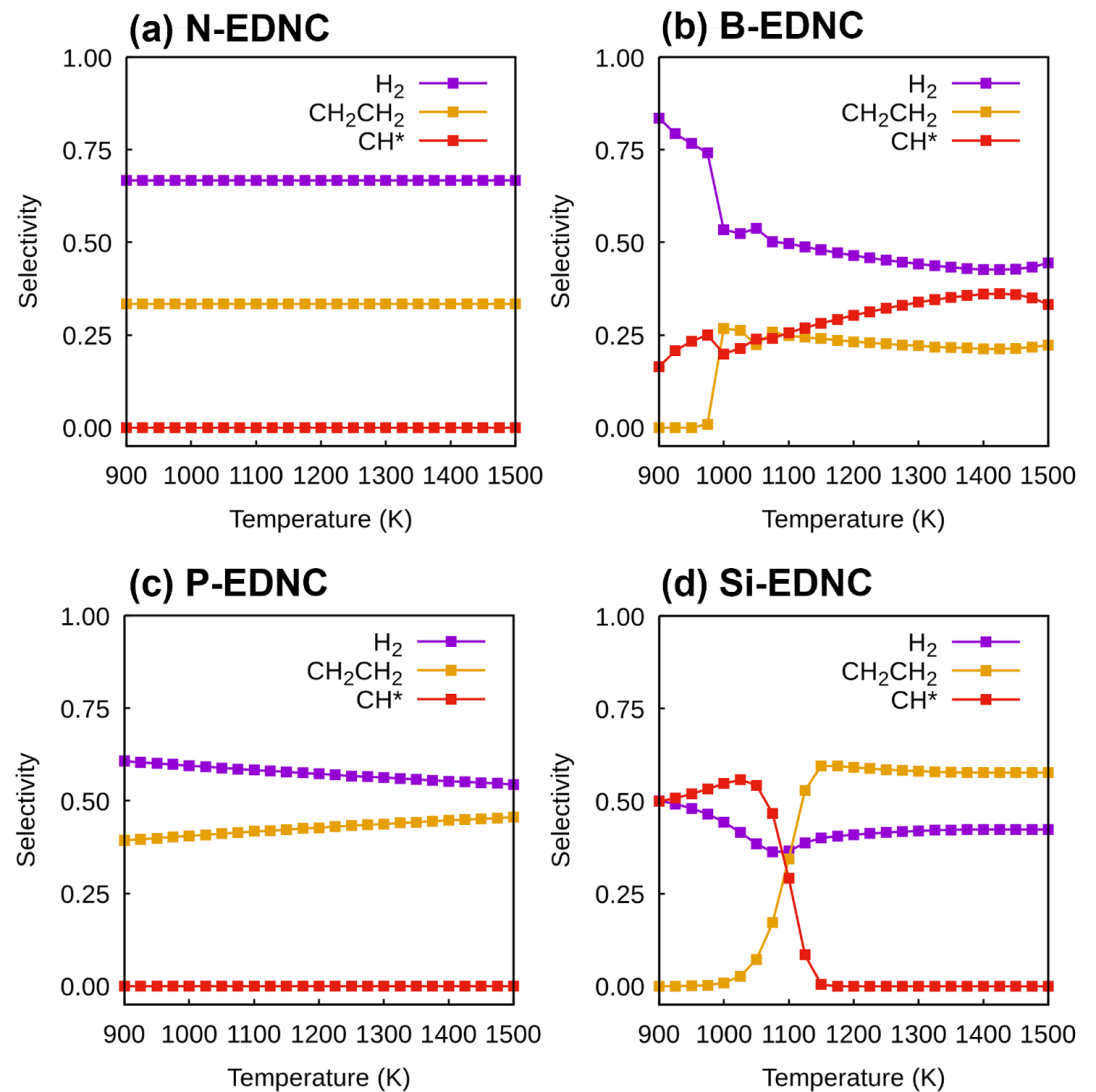
Selectivities to the production of H_2_, CH_2_CH_2_ and CH* on (a) N-EDNC, (b) B-EDNC, (c) P-EDNC and (d) Si-EDNC at the temperature range between 900 and 1500 K.

Comparison among product selectivities on EDNCs is reported in Figure 6. It was observed that the selectivities of H_2_, CH_2_CH_2_ and CH* formation remained the same in the entire temperature range studied here on N-EDNC, with highest selectivity for H_2_ formation. As shown in Figure 5, the formation of ethene is preferred over the CH_2_* dehydrogenation step on N-EDNC and P-EDNC by having free energies of activation 0.48 and 2.12 eV lower. On P-EDNC, the selectivity of CH_2_CH_2_ formation increases at higher temperatures as the rate coefficient ratio, , increases from 0.682 at 1000 K to 0.837 at 1500 K (Figure 6). The dehydrogenation of CH_2_* into CH* was more favorable on B-EDNC and Si-EDNC, with Δ*G*_*a*_ 2.66 and 1.70 eV lower, respectively, in comparison with the CH_2_* coupling step on the same EDNCs (Figure 5). The B-EDNC is most susceptible to coke formation among the EDNCs investigated here, confirmed by the highest selectivity of CH* formation (Figure 6b) and increasing coverage of CH* at higher temperatures (Figure S8). The coke formation is likely to occur at higher temperatures on the Si-EDNC catalyst; however, in Figure 6, it is possible to observe that the CH* selectivity decreases and CH_2_CH_2_ selectivity increases with temperature since the latter becomes kinetically feasible at high temperatures, with the ratio of rate coefficients of the reactions R9-Si (CH_2_* + CH_2_* → CH_2_CH_2_ + 2*) and R11-Si (CH_2_* + * → CH* + H*) increasing 3 orders of magnitude between 1000 and 1500 K.

## 4 Conclusion

In this work, we used DFT to investigate the mechanism of hydrogen production from methane decomposition on edge-decorated nanocarbons. After a systematic thermodynamic and microkinetic analysis, we concluded that nitrogen decoration on nanocatalyst edges (N-EDNC) presented an outstanding performance for H_2_ production at the temperature range between 900 and 1500 K, studied here, closely followed by phosphorus decoration (P-EDNC) at temperatures higher than 1200 K. Decorations by silicon (Si-EDNC) and boron (B-EDNC) were less efficient for activating hydrogen production, in comparison with N-EDNC and P-EDNC, as evidenced by the calculated turnover frequencies between 900 and 1500 K. When considering an atmosphere of atomic hydrogen on the simulations, the P-EDNC and Si-EDNC presented a superior performance for H_2_ production, in the partial pressure of H range between 10^–9^ bar and 10^–2^ bar, at 1000 K. Furthermore, P-EDNC presented the best activity for CH_2_* coupling into CH_2_CH_2_, contributing to the regeneration of the active sites on the surface, being critical for the continuity of the catalytic mechanism. The resistance of the EDNCs to coke deposition on the nanocarbon edges was investigated by considering the dehydrogenation of CH_2_* and its selectivity among the production of CH_2_CH_2_ and H_2_. We concluded that P-EDNC and N-EDNC have higher resistance to coke deposition, mainly due to their higher energy barrier for CH* formation and lower energy barrier for CH_2_CH_2_ formation. Although a good performance for methane activation was observed on B-EDNC, it showed lower resistance to coke formation at higher temperatures. On the other hand, the selectivity for catalytic sites’ regeneration on Si-EDNC increased at higher temperatures, being more resistant to coke formation. In conclusion, our results, combined with recent advances in the synthesis of extended heteroatom Klein-type edges on graphene, give us high expectations for the application of EDNCs as catalysts. Furthermore, the results presented in this study confirm that graphene-based materials can be efficiently employed as metal-free catalysts for the direct conversion of alkanes into high-value chemicals. Based on the outstanding performance of EDNCs presented here, we aim to explore these materials as electrocatalysts in future works, as the decorated edges might reduce the recombination of charge carriers and enhance the catalytic activity of decorated carbons.

## Supporting Information Available

AIMD simulations and discussion of the thermal stability of EDNCs, representation of the surface structures mentioned in the text, surface coverage as a function of temperature, reaction profile of ethane formation, adsorption energy values for CH_3_ and H, Mulliken atomic charges’ population, and Arrhenius parameters for the reactions included in the microkinetic simulations. (PDF)
