# Supplementary material for: First-Principles Microkinetic Modeling Unravelling the Performance of Edge-Decorated Nanocarbons for Hydrogen Production from Methane
Source: ACS Appl Mater Interfaces. 2023 Jan 26;15(5):6951–62. doi: 10.1021/acsami.2c20937 (PMC9923683; doi:10.1021/acsami.2c20937)
Supplement: Supplementary file 1 — am2c20937_si_001.pdf [file am2c20937_si_001.pdf]

Supporting Information

First-Principles Microkinetic Modelling Unravelling the Performance of Edge-Decorated Nanocarbons for Hydrogen Production from Methane

Neubi F. Xavier,[1,*] Jr., Glauco F. Bauerfeldt[2] and Marco Sacchi[1,**]

[1] School of Chemistry and Chemical Engineering, University of Surrey, Guildford, GU2 7XH, Surrey, UK

[2] Instituto de Química , Universidade Federal Rural do Rio de Janeiro, CEP 23890-000, Seropédica, Brazil

E-mail: * n.xavier@surrey.ac.uk; ** m.sacchi@surrey.ac.uk

Thermal Stability of EDNCs at reaction condition.

AIMD simulations were performed for each EDNC to further prove the stability of the catalysts. Simulations were performed at 1000 K for a time period of 5 ps, with a time step of 1 fs. Variation of energy per atom against time for the simulations for N-EDNC, B-EDNC, P-EDNC and Si-EDNC are shown in Figure S1. Snapshot of the initial and after 5 ps are represented in Figure R2b. At the end of the simulations, the structures of the catalysts remained similar to the initial structure with low energetic variation during the simulation, indicating that all EDNCs investigated here are stable at high temperatures.

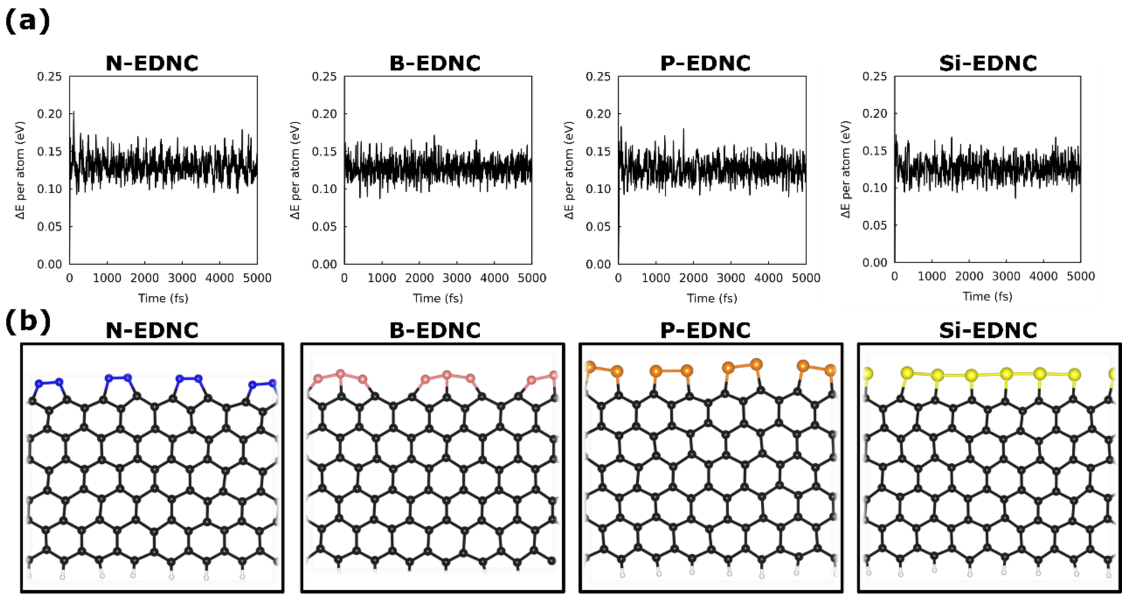

Figure S1. (a) Variation of electronic energy per atom, in respect to the minimum energy structure, against the time, upon AIMD simulations at 1000 K for N-EDNC, B-EDNC, P-EDNC and Si-EDNC. (b) Snapshot of the each EDNC structure after 5 ps simulation time.

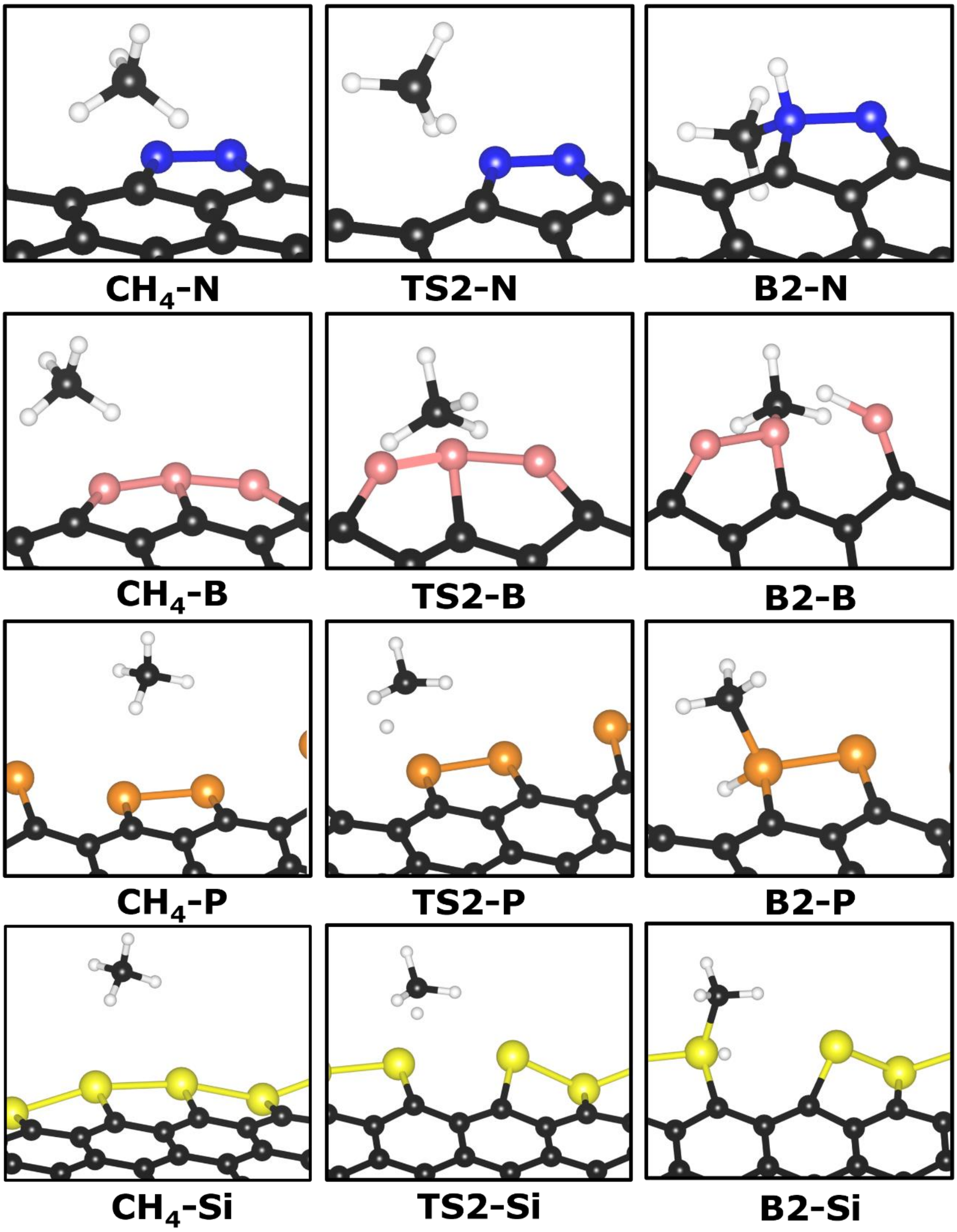

Figure S2. Geometries of the stationary points for the dissociative adsorption of methane into CH_3 and H chemisorbed on the same decorated atom.

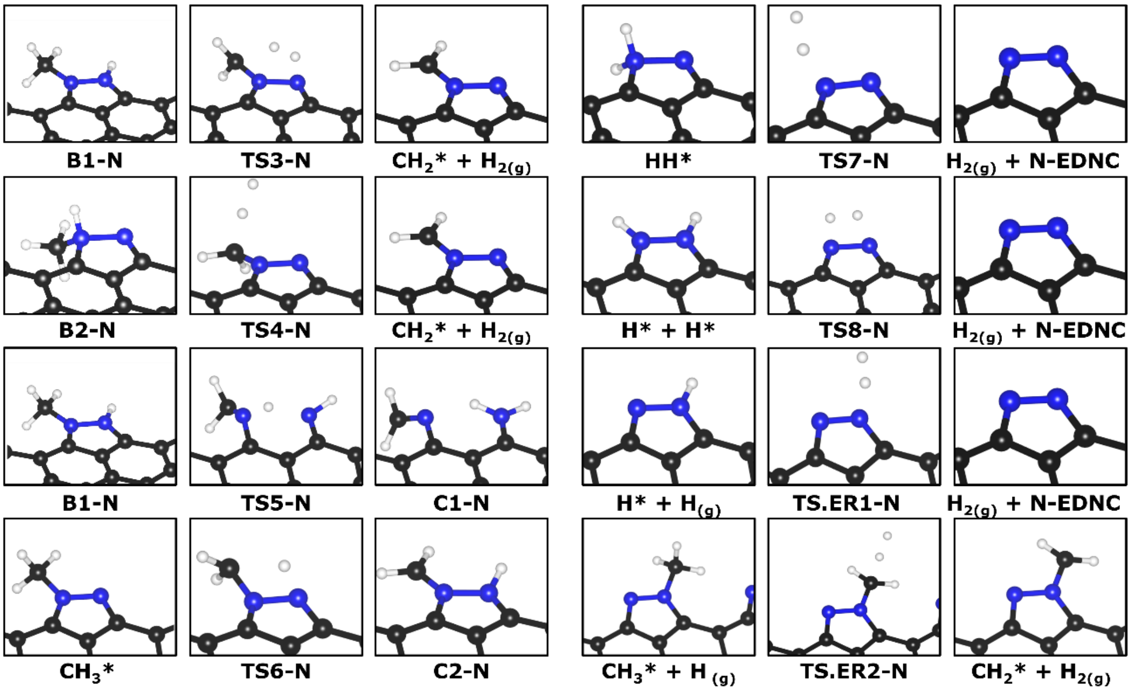

Figure S3. Geometries of the stationary points for the elementary reactions of the mechanism of H_2 formation on N-EDNC.

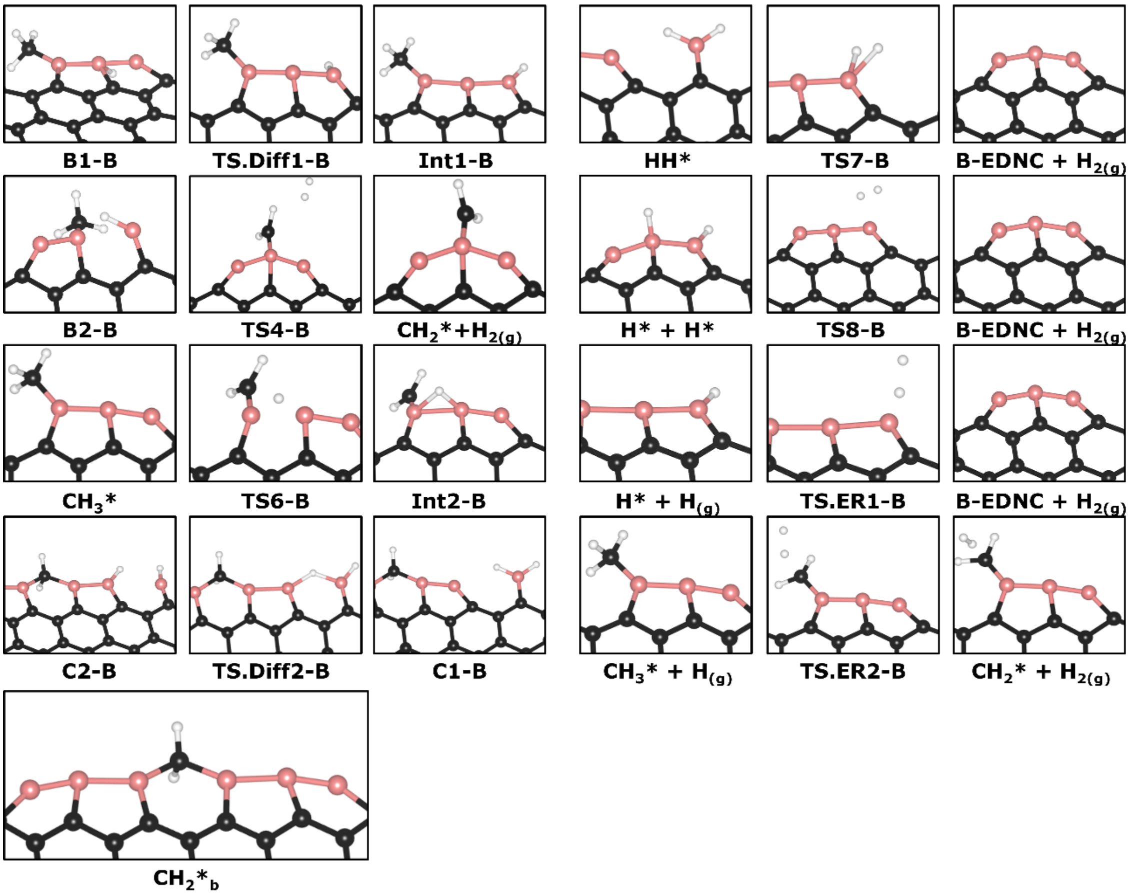

Figure S4. Geometries of the stationary points for the elementary reactions of the mechanism of H_2 formation on B-EDNC.

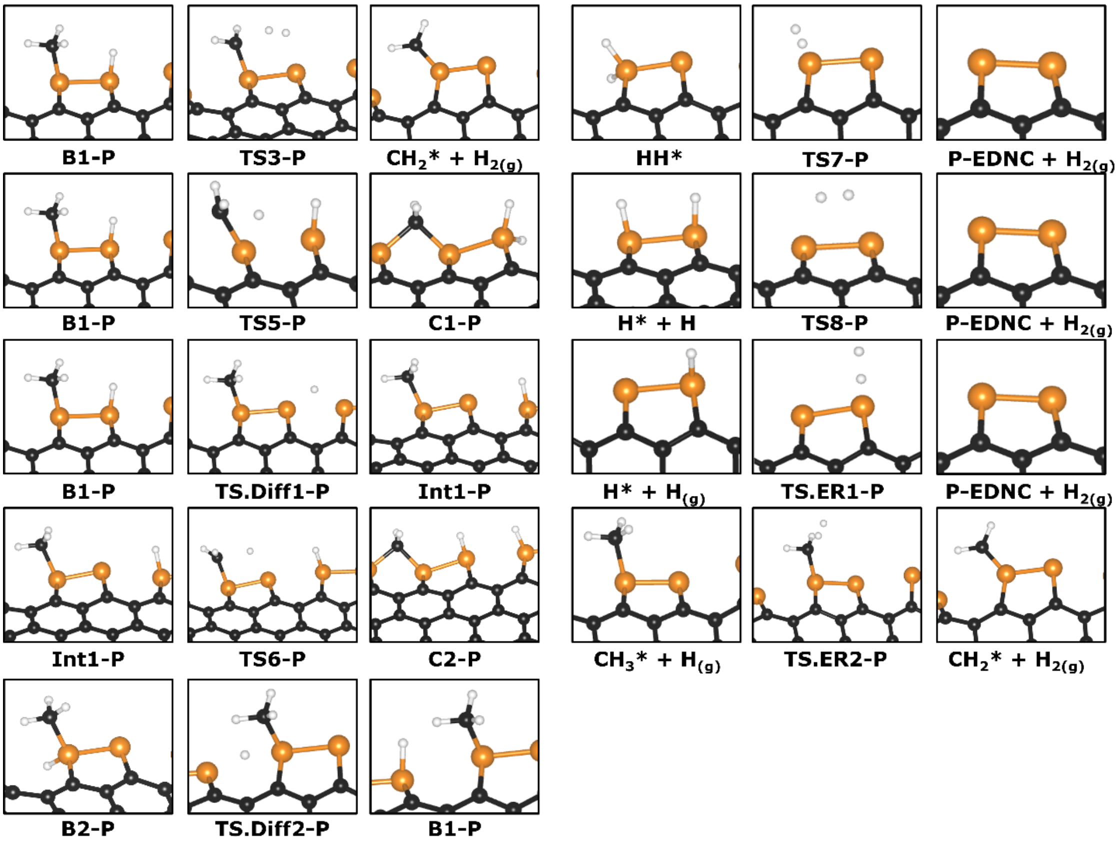

Figure S5. Geometries of the stationary points for the elementary reactions of the mechanism of H_2 formation on P-EDNC.

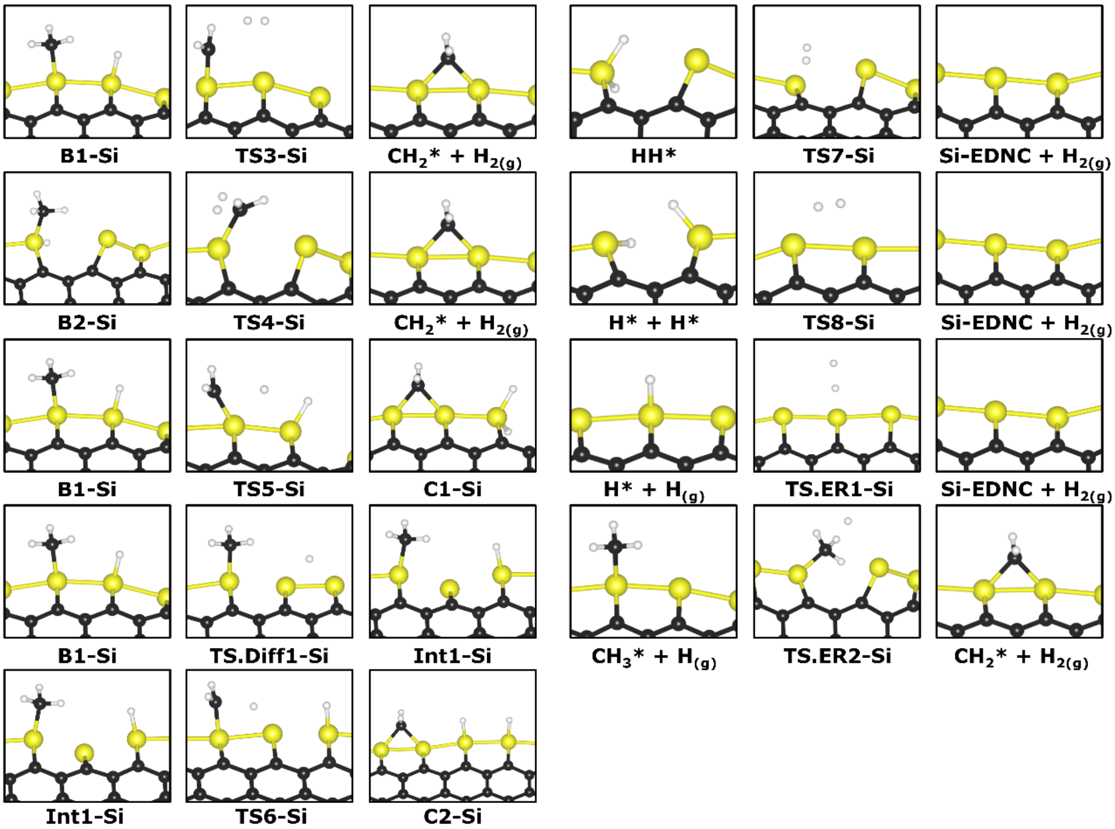

Figure S6. Geometries of the stationary points for the elementary reactions of the mechanism of H_2 formation on Si-EDNC.

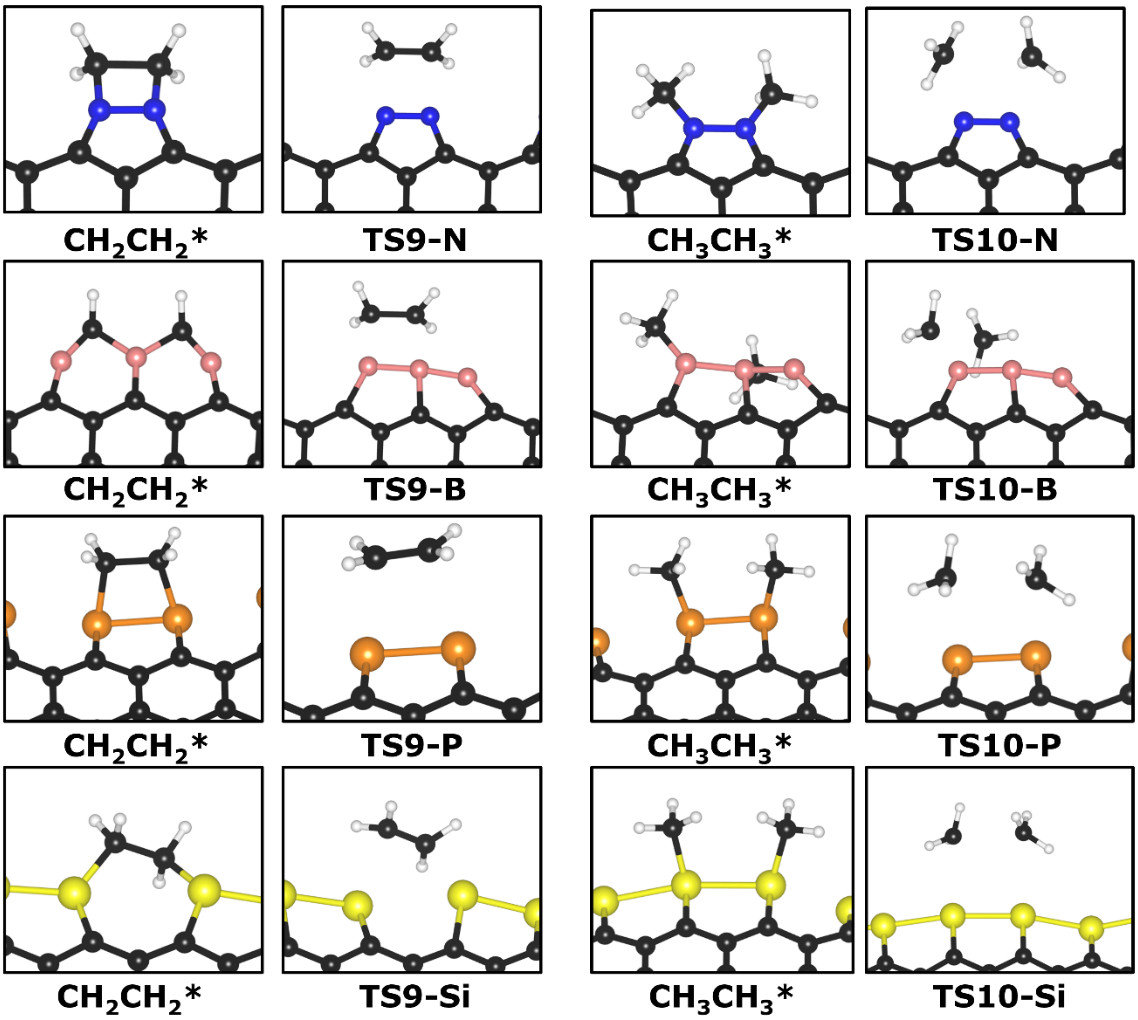

Figure S7. Geometries of the stationary points for the reaction steps of CH_2CH_2 and CH_3CH_3 formation on N-EDNC, B-EDNC, P-EDNC and Si-EDNC.

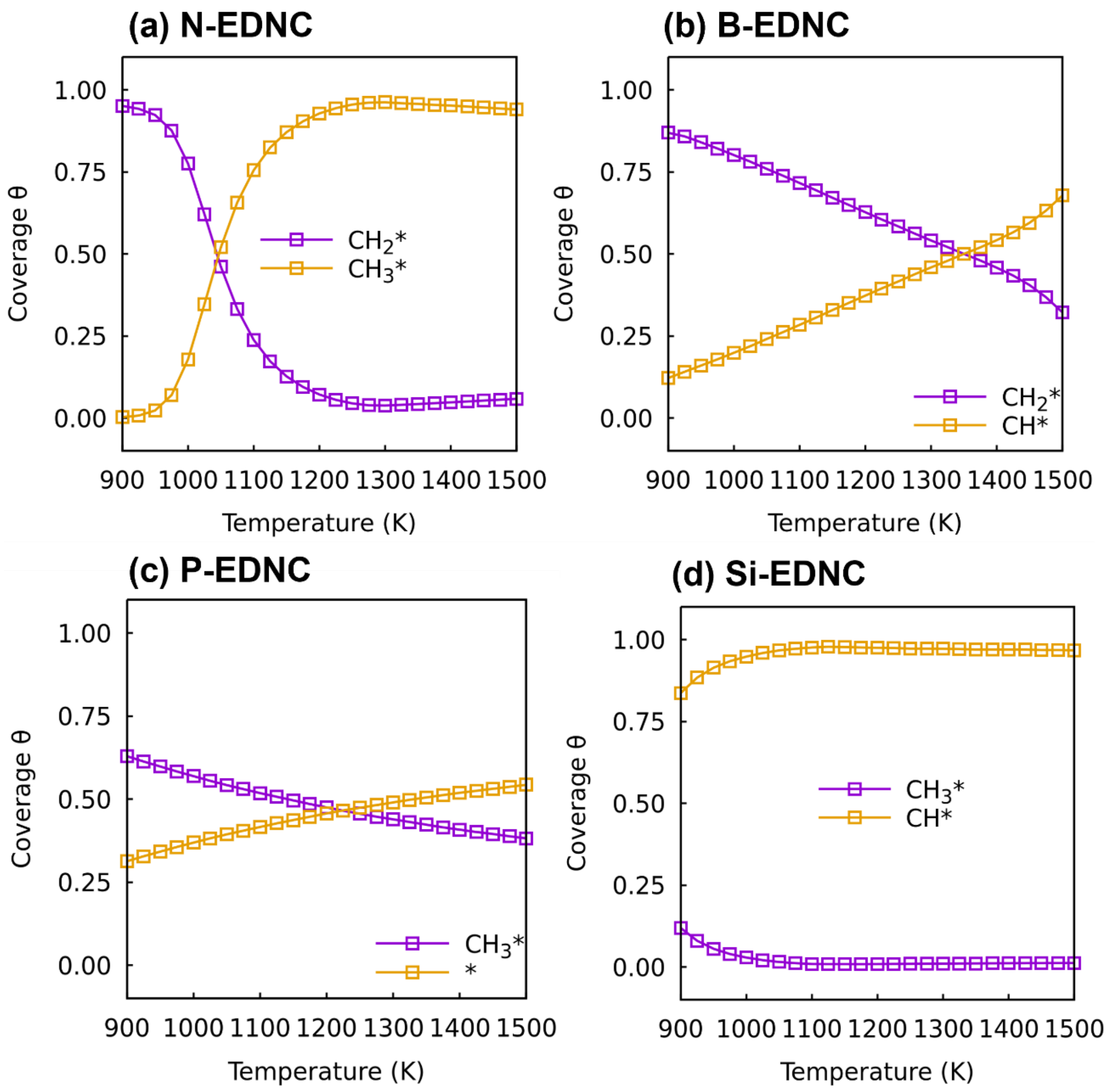

Figure S8. Surface coverage of surface species and free sites on (a) N-EDNC, (b) B-EDNC, (c) P-EDNC and (d) Si-EDNC.

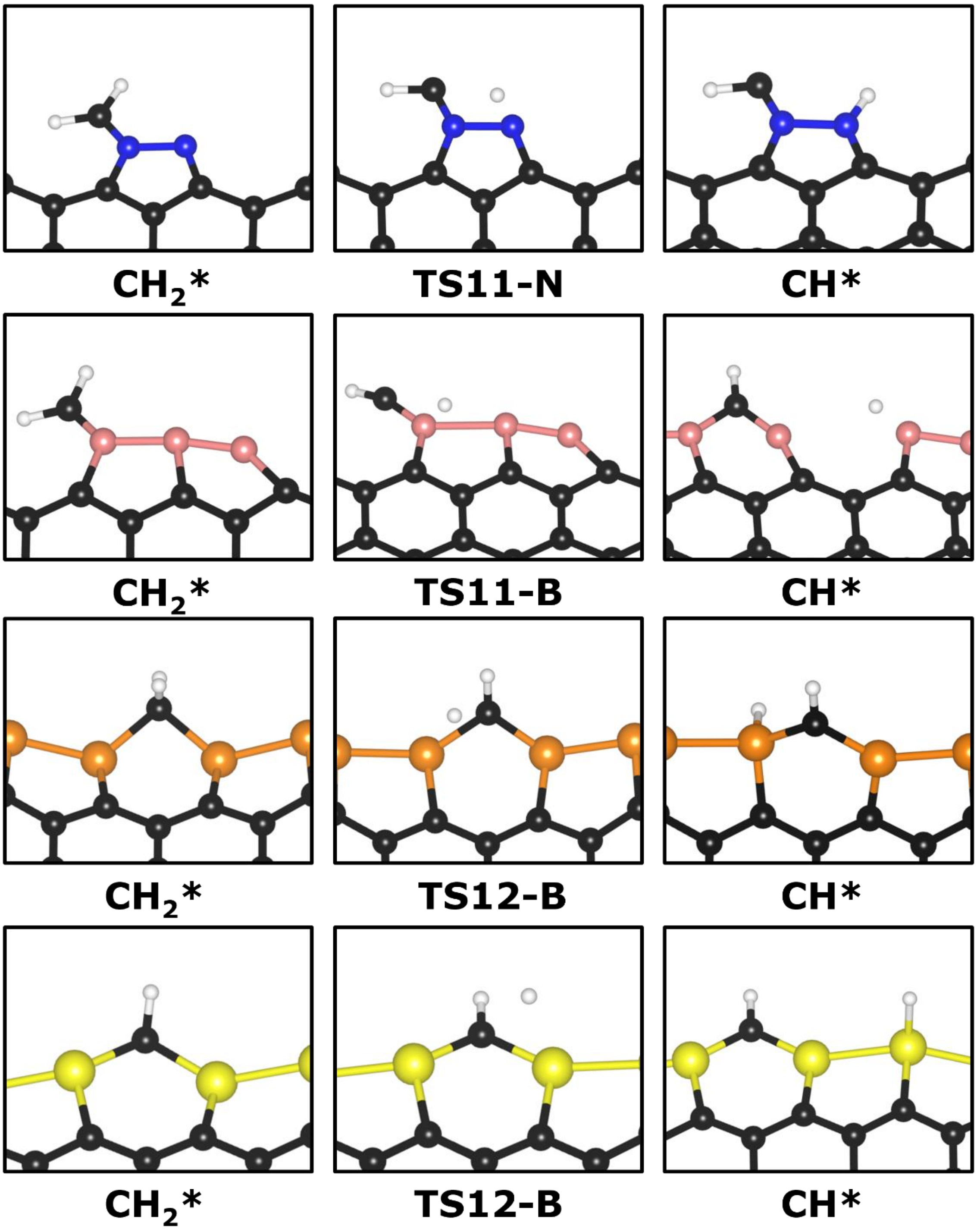

Figure S9. Geometries of the stationary points for the reaction steps of CH_2* decomposition into CH* on N-EDNC, B-EDNC, P-EDNC and Si-EDNC.

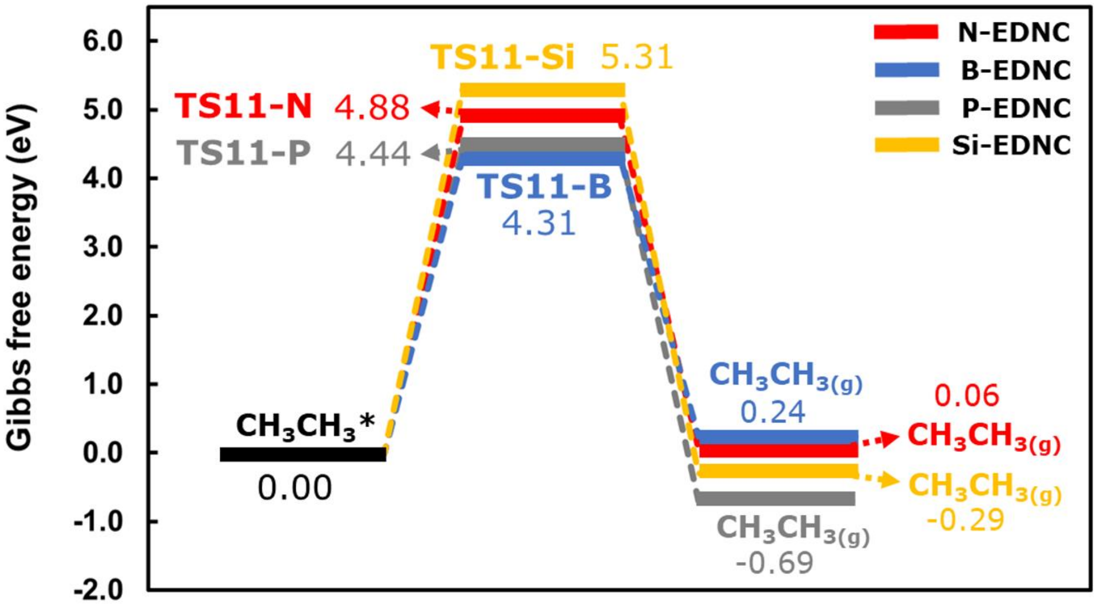

Figure S10. Reaction profile for the formation of CH_3CH_3 on N-EDNC, B-EDNC, P-EDNC and Si-EDNC.

Table S1. Adsorption energy (E_{ads}) (in eV) for the hydrogen atom and methyl chemisorbed on N-EDNC, B-EDNC, P-EDNC and Si-EDNC. All values are reported in eV.

	N-EDNC	B-EDNC	P-EDNC	Si-EDNC
E_{ads} (H)	-2.21809	-1.31466	-0.63257	-0.90201
E_{ads} (CH_3)	-1.4982	-1.25027	-0.20168	-0.58177

Table S2. Mulliken atomic charges population, in units of electrons (e), for the decorated atom on the nanocarbon edge and considering the chemisorption of CH_3 and H on the EDNC edges

<table>
<tr><th></th><th>Y=H</th><th>Y=CH₃</th><th>ΔQ (H)</th><th>ΔQ (CH₃)</th></tr>
<tr><td>N</td><td>-0.211</td><td>-0.213</td><td rowspan="2">-0.182</td><td rowspan="2">0.083</td></tr>
<tr><td>N-Y</td><td>-0.393</td><td>-0.130</td></tr>
<tr><td>B</td><td>-0.004</td><td>0.191</td><td rowspan="2">-0.190</td><td rowspan="2">0.224</td></tr>
<tr><td>B-Y</td><td>-0.194</td><td>0.415</td></tr>
<tr><td>P</td><td>0.200</td><td>0.187</td><td rowspan="2">0.037</td><td rowspan="2">0.260</td></tr>
<tr><td>P-Y</td><td>0.237</td><td>0.447</td></tr>
<tr><td>Si</td><td>0.332</td><td>0.310</td><td rowspan="2">0.048</td><td rowspan="2">0.380</td></tr>
<tr><td>Si-Y</td><td>0.380</td><td>0.69</td></tr>
</table>

Table S3. Kinetic parameters for the elementary reactions involved in the microkinetic modelling on N-EDNC.

		A (s^{-1})		Ea (eV)	
**Label**	**Reaction**	**fwd**	**bwd**	**fwd**	**bwd**
R1-N	CH4 + 2* → CH_3* + H*	1.32×10^{13}	1.14×10^{15}	1.49	3.45
R2-N	CH4 + * → CH_3-H*	1.40×10^{13}	2.04×10^{15}	0.95	2.93
R3-N	CH_3* + H* → CH_2* + H_2 + *	2.43×10^{14}	1.46×10^{12}	3.51	1.65
R4-N	CH_3-H* → CH_2* + H_2	3.93×10^{14}	8.07×10^{11}	1.96	1.23
R5-N	CH_3* + H* → CH_2* + HH*	2.96×10^{14}	1.46×10^{14}	3.12	3.45
R6-N	CH_3* + * → CH_2* + H*	5.67×10^{13}	2.26×10^{13}	2.89	2.06
R7-N	HH* → H_2 + *	1.03×10^{15}	4.21×10^{11}	1.98	0.65
R8	2H* → H_2 + 2*	7.81×10^{14}	5.51×10^{11}	4.12	1.52
R.ER1-N	H + H* → H_2 + *	2.74×10^{12}	1.34×10^{11}	0.76	0.86
R.ER2-N	H + CH_3* → H_2 + CH_2*	2.03×10^{13}	1.46×10^{13}	2.32	3.13
R.H-N	H* → H + *	1.91×10^{15}	5.47×10^{12}	4.29	0.01
R9-N	CH_2* + CH_2* → CH_2CH_2 + 2*	5.53×10^{14}	9.38×10^{11}	2.30	0.47
R10-N	CH_3* + CH_3* → CH_3CH_3 + 2*	2.20×10^{16}	3.55×10^{14}	5.49	3.47
R11-N	CH_2* + * → CH* + H*	4.78×10^{13}	3.77×10^{13}	2.57	1.17

Table S4. Kinetic parameters for the elementary reactions involved in the microkinetic modelling on B-EDNC.

		**A (s^{-1})**		**Ea (eV)**	
**Label**	**Reaction**	**fwd**	**bwd**	**fwd**	**bwd**
R1-B	CH4 + 2* → CH_3* + H*	3.14×10^{13}	8.66×10^{14}	2.48	4.47
R2-B	CH4 + * → CH_3-H*	4.20×10^{12}	3.28×10^{13}	1.22	1.54
R3-B	CH_3* + H* → CH_2* + H_2 + *	1.25×10^{14}	5.59×10^{11}	1.38	0.06
R4-B	CH_3-H* → CH_2* + H_2	9.96×10^{13}	3.97×10^{11}	2.56	0.98
R5-B	CH_3* + H* → CH_2* + HH*	5.44×10^{13}	2.65×10^{13}	1.56	1.05
R6-B	CH_3* + * → CH_2* + H*	5.44×10^{13}	2.65×10^{13}	1.56	1.05
R7-B	HH* → H_2 + *	1.25×10^{14}	5.59×10^{11}	1.38	0.06
R8	2H* → H_2 + 2*	4.32×10^{13}	1.58×10^{11}	2.69	0.62
R.ER1-B	H + H* → H_2 + *	1.14×10^{13}	2.15×10^{12}	0.66	2.17
R.ER2-B	H + CH_3* → H_2 + CH_2*	1.50×10^{13}	8.90×10^{11}	2.70	3.36
R.H-B	H* → H + *	3.01×10^{14}	5.35×10^{12}	3.07	0.23
R9-B	CH_2* + CH_2* → CH_2CH_2 + 2*	1.01×10^{15}	3.12×10^{13}	5.47	1.40
R10-B	CH_3* + CH_3* → CH_3CH_3 + 2*	5.92×10^{14}	3.00×10^{14}	4.74	4.37
R11-B	CH_2* + * → CH* + H*	7.29×10^{14}	2.73×10^{15}	3.33	2.13

Table S5. Kinetic parameters for the elementary reactions involved in the microkinetic modelling on P-EDNC.

		A (s^{-1})		Ea (eV)	
**Label**	**Reaction**	**fwd**	**bwd**	**fwd**	**bwd**
R1-P	CH4 + 2* → CH_3* + H*	1.33×10^{13}	3.45×10^{14}	2.53	2.76
R2-P	CH4 + * → CH_3-H*	4.42×10^{12}	1.38×10^{14}	2.46	2.68
R3-P	CH_3* + H* → CH_2* + H_2 + *	1.02×10^{14}	1.97×10^{11}	2.61	0.62
R4-P	CH_3-H* → CH_2* + H_2	1.02×10^{14}	1.97×10^{11}	2.61	0.62
R5-P	CH_3* + H* → CH_2* + HH*	7.76×10^{13}	1.66×10^{14}	2.64	2.38
R6-P	CH_3* + * → CH_2* + H*	6.45×10^{13}	1.31×10^{14}	2.74	2.60
R7-P	HH* → H_2 + *	4.15×10^{14}	3.36×10^{11}	2.71	2.17
R8	2H* → H_2 + 2*	1.49×10^{14}	7.51×10^{11}	2.33	1.49
R.ER1-P	H + H* → H_2 + *	4.12×10^{12}	3.83×10^{11}	0.65	2.41
R.ER2-P	H + CH_3* → H_2 + CH_2*	1.30×10^{12}	1.92×10^{11}	0.29	0.78
R.H-P	H* → H + *	3.09×10^{14}	8.73×10^{12}	2.62	0.01
R9-P	CH_2* + CH_2* → CH_2CH_2 + 2*	2.79×10^{14}	7.17×10^{12}	1.82	0.54
R10-P	CH_3* + CH_3* → CH_3CH_3 + 2*	1.00×10^{15}	8.87×10^{14}	4.79	4.42
R11-P	CH_2* + * → CH* + H*	1.54×10^{14}	5.25×10^{13}	3.31	2.17

Table S6. Kinetic parameters for the elementary reactions involved in the microkinetic modelling on Si-EDNC

		**A (s^{-1})**		**Ea (eV)**	
**Label**	**Reaction**	**fwd**	**bwd**	**fwd**	**bwd**
R1-Si	CH4 + 2* → CH_3* + H*	1.88×10^{12}	5.40×10^{13}	0.92	2.05
R2-Si	CH4 + * → CH_3-H*	2.72×10^{12}	1.92×10^{14}	1.82	2.84
R3-Si	CH_3* + H* → CH_2* + H_2 + *	3.25×10^{15}	9.64×10^{12}	2.81	1.70
R4-Si	CH_3-H* → CH_2* + H_2	4.79×10^{12}	3.68×10^{12}	3.87	3.68
R5-Si	CH_3* + H* → CH_2* + HH*	2.82×10^{14}	1.28×10^{15}	3.57	3.66
R6-Si	CH_3* + * → CH_2* + H*	1.87×10^{13}	7.01×10^{13}	1.90	1.93
R7-Si	HH* → H_2 + *	2.03×10^{14}	1.10×10^{11}	2.67	1.64
R8	2H* → H_2 + 2*	2.57×10^{14}	6.32×10^{11}	1.54	0.71
R.ER1-Si	H + H* → H_2 + *	4.92×10^{13}	5.83×10^{12}	2.35	3.95
R.ER2-Si	H + CH_3* → H_2 + CH_2*	1.56×10^{13}	4.83×10^{11}	0.90	3.65
R.H-Si	H* → H + *	2.59×10^{14}	5.19×10^{12}	2.80	0.02
R9-Si	CH_2* + CH_2* → CH_2CH_2 + 2*	3.16×10^{15}	2.31×10^{13}	3.77	1.81
R10-Si	CH_3* + CH_3* → CH_3CH_3 + 2*	1.99×10^{15}	1.45×10^{15}	5.72	4.24
R11-Si	CH_2* + * → CH* + H*	3.19×10^{13}	2.44×10^{13}	1.67	2.00
